# Smooth muscle of the lower urinary tract: BK-RyR coupling in physiology and pathophysiology

**DOI:** 10.1007/s10974-025-09707-w

**Published:** 2025-09-01

**Authors:** Monica Ridlon, Julia Tlapa, Kimberly Keil Stietz

**Affiliations:** Department of Comparative Biosciences, University of Wisconsin-Madison, 2015 Linden Dr, Madison, WI 53706 USA

**Keywords:** Smooth muscle contraction, Lower urinary tract symptoms

## Abstract

In the lower urinary tract, coordinated function between the bladder and urethra is essential for normal micturition, requiring smooth muscle contraction and relaxation in a tightly regulated cycle. During the bladder filling phase, the bladder remains relaxed while the urethra stays contracted to prevent leakage. During voiding, this coordination reverses, and the bladder contracts to expel urine while the relaxed urethra allows urine flow. These functions are essential for proper micturition and two key molecular regulators of this process are the large-conductance calcium activated potassium (BK) channels and ryanodine receptors (RyRs), both of which modulate smooth muscle excitability and calcium dynamics. This review provides an overview of the roles of BK channels and RyR mediated signaling in regulating smooth muscle activity in the bladder and urethra, with a focus on their contributions to lower urinary tract physiology and pathophysiology. BK channels act as negative feedback modulators, dampening myogenic and nerve-evoked contractions of the detrusor and urethra. RyRs play a role in regulating intracellular calcium signaling that supports both muscle relaxation and contraction. This review highlights alterations in the function of these channels in lower urinary tract dysfunction, and as potential targets for other factors such as environmental exposures to disrupt voiding function. BK channels and RyRs are plausible targets for therapeutic strategies aimed at improving bladder and urethra function in certain patients, particularly those with lower urinary tract symptoms caused by factors such as aging and environmental chemical exposure.

## Introduction

The bladder and urethra work in tandem to properly store and excrete urine in the healthy micturition cycle. When focusing on the lower urinary tract, the healthy micturition cycle can be split into two phases, bladder filling and bladder emptying. During the bladder filling phase, the bladder is relaxed and stretches to comply with increased urine volume (Chakrabarty et al. 2019; Drake et al. 2017), while the urethral sphincter generates sufficient contractile force to maintain continence and prevent urinary leakage (DeLancey et al. 2008; Jung et al. 2012). During the voiding phase, the bladder and urethra coordinate to swap contractile states such that the bladder contracts while the urethral sphincter relaxes to expel urine (Heesakkers and Gerretsen 2004). The proper function of these two tissues, especially in relation to control of smooth muscle contraction, is vital to regulate micturition.

Dysfunction of the bladder or urethra can give rise to burdensome lower urinary tract symptoms. While there are numerous risk factors such as genetic mutations, environmental insults, comorbid health conditions and aging that can contribute to the onset, progression, and severity of lower urinary tract symptoms, this review will examine insults targeting the smooth muscle layer of the bladder and urethra with a focus on large-conductance (100–300 pS) voltage- and Ca^2+^-activated K + channels (BK, BK_Ca_, Maxi-K, *Slo1*, or K_Ca_1.1) and ryanodine-sensitive Ca^2+^ release channels (ryanodine receptors, RyRs) (Yoshida et al. 2001). Together, BK channels and RyRs contribute in part to the regulation of smooth muscle tone of both the bladder and urethra. Although drug therapies for lower urinary tract symptoms do not currently target BK channels and RyRs, this review aims to outline their role in lower urinary tract physiology and pathophysiology to highlight how these ion channels can drive lower urinary tract dysfunction in preclinical models and be a target for novel therapeutic approaches to improve urinary tract function.

## Lower urinary tract physiology

Bladder wall histology is primarily segmented into 4 layers: urothelium, lamina propria, smooth muscle, and serosa/adventitia (Bolla et al. 2025). Briefly, starting at the lumen, the urothelium consists of several cell populations including basal, intermediate, and superficial cells. This superficial layer consists of specialized umbrella cells whose surfaces are covered by urothelial plaques composed of uroplakins, which provide barrier function (Wu et al. 2009). The lamina propria separates the urothelium from the detrusor with a layer of extracellular matrix and several cell types including fibroblasts, adipocytes, interstitial cells of Cajal, nerves, and blood vessels (Andersson and McCloskey 2014). The smooth muscle layer of the bladder, known as the detrusor, consists of myocyte fascicles arranged in spherical bundles in the central section of the detrusor with longitudinal layers on either side (Christ and Hodges 2006; Kuijpers et al. 2014). As the bladder fills, detrusor tone is maintained through bladder wall stretching and thinning where the myocytes lengthen (Uvelius and Gabella 1980). Lastly, the serosa and adventitia form connective tissue layers around the bladder and connect it to the peritoneal layer of the abdominal wall (Bolla et al. 2025).

Urethral histology is segmented into the same 4 layers as the bladder, with differences in cellular composition that vary in relation to sex and proximity to the bladder; Henry et al. 2018; Hudson et al. 2001). For example, the urethral urothelium of the prostatic urethra includes additional club and hillock epithelial cells (Henry et al. 2018) and the lamina propria has additional glands in the prostatic urethra and near the female external urethral sphincter (Eggermont et al. 2019; Satoh et al. 2001; Stoddard et al. 2025; Zaviacic et al. 1997). The smooth muscle layer, separated from the lamia propria by a thin fascial layer, is organized into an inner layer of longitudinal smooth muscle and outer layer of circular smooth muscle (Abelson et al. 2018). Interspersed within the urethra smooth muscle layer are electrically-coupled interstitial cells of Cajal which are involved in the pacemaker activity of urethral contraction during homeostatic conditions (Sergeant et al. 2000). The role of urethral interstitial cells of Cajal was recently expanded to include driving a contractile response to expel pathogens, such as defending against *E. coli* ascension in a mouse model of urinary tract infection (Ambrogi et al. 2025). The smooth muscle cells of the internal urethral sphincter are primarily responsible for autonomic, involuntary continence while the external sphincter is composed of striated (skeletal) muscle and is responsible for somatic, voluntary control of continence (Greenland et al. 1996; Rother et al. 1996). While the external sphincter plays an important role in voluntary “contingency” continence (such as during coughing, sneezing, or laughter), this review will focus on the smooth muscle and internal urethral sphincter. (Ferreira and Duarte Cruz 2021). Both the internal and external sphincters are necessary for the regulation of micturition, functioning together to regulate voiding in coordination with the bladder (Brading 1999; Greenland et al. 1996; Koraitim 2008).

## Innervation and smooth muscle contraction

**Fig. 1:**
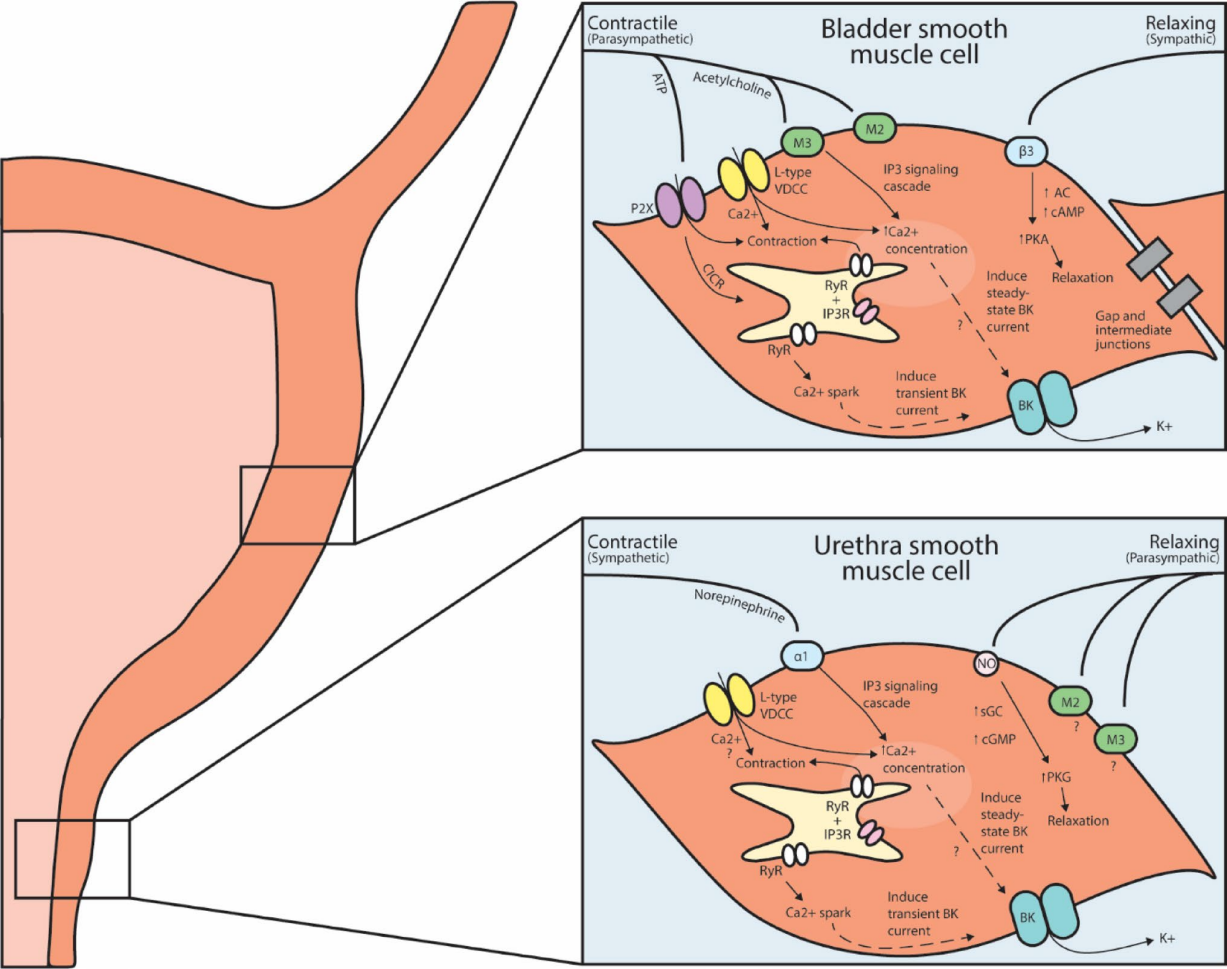
Representation of pathways involved in the contraction and relaxation of bladder and urethra smooth muscle, including relevant neurotransmitters and receptors involved in signaling. ATP: adenosine triphosphate; M_2_/M_3_: muscarinic receptors; L-type VDCC: L-type voltage dependent calcium channels; P2X: purinergic receptor; RyR: ryanodine receptor; IP_3_R: inositol triphosphate receptor; PKA, PKG: protein kinase A, G; AC: adenylyl cyclase; cAMP: cyclic adenosine monophosphate; sGC: soluble guanylate cyclase; cGMP: cyclic guanosine monophosphate; BK: big potassium channel; Ca^2+^: calcium

### Bladder filling

The average human bladder holds around 500 mL of urine at full capacity, but does not reach full capacity in healthy conditions before a urination event occurs, and healthy individuals hold conscious control over when and where to urinate (Griffiths and Tadic 2008; Kershen et al. 2002). During the bladder filling phase (Fig. 1), lumbar sympathetic fibers travelling via the hypogastric nerves release norepinephrine, which binds to beta 3-adrenergic receptors (β3) on detrusor smooth muscle cells. This activates adenylate cyclase, increasing cAMP levels and activating protein kinase A, which phosphorylates various downstream targets leading to detrusor relaxation. One downstream effect also includes the activation of transient BK channel currents (TBKCs), which contribute to membrane hyperpolarization and reduced excitability (Michel and Vrydag 2006; Petkov and Nelson 2005). In the proximal urethra, sympathetic activity excites smooth muscle via release of norepinephrine, which binds primarily to α1-adrenoceptors, ultimately leading to smooth muscle contraction and closure of the internal sphincter to maintain continence during bladder filling. During this period, intracellular Ca^2+^ waves support sustained smooth muscle contraction. As part of the repolarization phase, BK channels open in response to elevated intracellular Ca^2+^, promoting membrane hyperpolarization, which limits excessive excitability and contributes to rhythmic control of contractile tone (Drumm et al. 2024; Kunisawa et al. 1985; Kyle et al. 2013; Michel and Vrydag 2006; Ueda et al. 1984). The role of voltage-dependent calcium channels (VDCCs) in urethral smooth muscle contraction is not fully defined and may vary across species. In murine studies, L-type VDCCs do not appear to be essential for contraction (Drumm et a. 2018). However, studies in pig, rabbit, and human urethra have shown that L-type VDCC mediated Ca^2+^ influx is necessary to trigger transient BK currents and contractile response to adrenergic and purinergic stimuli (Brading et al. 1996; Teramoto and Brading 1996; Kyle et al. 2013). Species and sex differences in adrenoceptor expression/function have also been reported and are an area of active study (Hsieh et al. 2023). In addition to adrenergic signaling, acetylcholine release from parasympathetic nerve fibers may also contribute to urethral contraction via muscarinic receptors, mainly M_2_ and M_3_ subtypes, though their exact roles and functional significance remain to be fully elucidated (Mutoh et al. 1997; Nagahama et al. 1998). Somatic efferent activity via the pudendal nerve leads to release of acetylcholine, which binds nicotinic receptors on striated muscle of the external urethral sphincter leading to voluntary contraction important for continence (Damaser et al. 2003; Kerns et al. 2000).

### Bladder emptying

During bladder emptying (Fig. 1), efferent parasympathetic fibers from the pelvic nerve release acetylcholine and adenosine triphosphate (ATP), which trigger detrusor smooth muscle contraction via activation of muscarinic acetylcholine receptors and purinergic receptors, respectively (Brading 2002; Chess-Williams 2002; Daniel et al. 1983; Giglio and Tobin 2009; Malysz and Petkov 2020). Purinergic signaling largely through P2X receptors, causes rapid depolarization and transient contractions, while cholinergic signaling, primarily via M_3_ muscarinic receptors, is responsible for the more sustained, tonic contraction necessary for voiding (Heppner et al. 2009). Although M_2_ muscarinic receptors are more abundant than M_3_ in detrusor, they do not directly mediate contraction, instead they likely contribute indirectly by opposing β-adrenergic mediated relaxation through inhibition of adenylyl cyclase, or by modulating M_3_ receptor mediated responses (Chapple et al. 2002; Yamanishi et al. 2001). Membrane depolarization activates downstream calcium signaling pathways. Membrane depolarization opens L-type VDCCs, which permits Ca^2+^ influx. Additionally, intracellular Ca^2+^ is released from the sarcoplasmic reticulum through IP_3_ receptors and RyR, amplifying the contractile response (Brading 2002; Daniel et al. 1983; Gong et al. 2021; Malysz and Petkov 2020). Detrusor smooth muscle cells are interconnected by gap junctions, enabling electrical coupling and coordinated contraction, but this coupling is less extensive than in other smooth muscle tissues (Clapham 1996).

In the urethra parasympathetic nerve terminals release nitric oxide and acetylcholine to relax urethral smooth muscle (Andersson et al. 1991, 1992). Once nitric oxide crosses the smooth muscle membrane, it activates soluble guanylate cyclase, which increases the production of cyclic GMP (cGMP). cGMP then activates protein kinase G (PKG), which inhibits Ca^2+^ release via the IP_3_ signaling cascade, ultimately leading to smooth muscle relaxation (Andersson et al. 1992). Purinergic signaling may also be involved in smooth muscle relaxation and contraction, though evidence is limited. ATP administration has been shown to cause both relaxation and contraction depending on urethral tone and ATP concentration (Callahan and Creed 1981; Sergeant et al. 2019). The purinergic signaling pathway remains unclear but may involve P2Y1, P2 × 1, or P2 × 2/3R receptors on the smooth muscle and/or interstitial cells (Bradley et al. 2011; Burnstock 2014; Salazar et al. 2019).

During bladder emptying, sympathetic efferent activity via the hypogastric nerves to the bladder and urethra is inhibited to allow detrusor contraction and internal urethral sphincter relaxation respectively. In addition, urethral somatic efferent activity via the pudendal nerve is also inhibited to permit relaxation of the external urethral sphincter (Damaser et al. 2003; Kerns et al. 2000).

## Pathophysiology

### Lower urinary tract symptoms and dysfunction

Lower urinary tract symptoms (LUTS) can be broadly characterized as symptoms related to urine storage, urinary voiding, or following urinary voiding (Abrams et al. 2003). The underpinning lower urinary tract dysfunction that drives these symptoms can be broadly characterized as failure to store urine - including detrusor overactivity, hypersensitivity, stress urinary incontinence, intrinsic sphincter deficiency, or failure to empty the bladder - including neurologic injury, detrusor underactivity, bladder outlet obstruction, urethral obstruction/stricture and striated sphincter dyssynergia (McDonough and Ryan 2016).

The onset of LUTS can be acquired through congenital anomalies, fetal or early life developmental disorders, injury throughout life, health conditions indirectly influencing lower urinary tract function (i.e. multiple sclerosis, diabetes, metabolic syndrome, obesity), or from aging-related processes including benign prostatic hyperplasia in men (Bharti et al. 2023; Kopač 2024; Lee and Kuo 2017; Moussa et al. 2020; Thomas et al. 2021). In a study based in Boston following a prospective cohort in the 2000’s, over 5 years moderate to severe LUTS were newly acquired in 11.4% of the individuals (Maserejian et al. 2013). The prevalence of LUTS has been increasing globally since the 1990 s, estimated at 45.2% in 2011, and in 2022 the estimated overall incidence of LUTS, and more specifically moderate-to-severe LUTS, is 63.2% and 31.3%, respectively (Huang et al. 2023; Irwin et al. 2011; Tahra et al. 2022).

The cost of LUTS is not solely attributed to medical care, of which the NIH estimates bladder, prostate and other urinary tract diseases cost Americans nearly $11 billion a year, and half this cost is covered through the social welfare program Medicare (National Institutes of Health (NIH), 2015). LUTS also drives an 8.03% greater overall work productivity loss and a decreased health-related quality of life (Kannan et al. 2009). For hospitalized patients, urinary incontinence is seen as a fate equal to or worse than death (Rubin et al. 2016). This observation underscores the importance of LUTS research, symptom prevention and modulation, as well as treatment.

### Over- and underactive bladder

The general clinical presentations of over- and underactive bladder (OAB and UAB) both include urgency and frequency, but UAB also includes weak stream, straining, hesitancy, and sensation of incomplete emptying (Foon and Toozs-Hobson 2007; Gormley et al. 2015; Hoag and Gani 2015). The prevalence of OAB in men and women ranges between 11.8% and 35.6% with increased incidence associated with age and body mass index (Eapen and Radomski 2016; Reynolds et al. 2016; Stewart et al. 2003). OAB is associated with diminished quality of life, quality of sleep, and mental health (Stewart et al. 2003). The prevalence of UAB is predicted to be under-reported as a 2014 study reported 23% of survey respondents reported sensation of incomplete bladder emptying, yet only 11% of survey respondents knew of UAB (Aggarwal and Zimmern 2016; Valente et al. 2014).

The pathophysiology of OAB and UAB are unclear, but changes to the bladder may include denervation in OAB and UAB, decreased bladder wall tone in OAB, and muscle loss and fibrosis in UAB (Brading 1997; Drake et al. 2017; Elbadawi et al. 1993; Rosenberg et al. 2016). The occurrence of OAB is often associated with detrusor overactivity with or without urgency incontinence, and UAB is often associated with detrusor underactivity (Chess-Williams and Sellers 2023; Osman et al. 2014).

### Detrusor over- and underactivity

Detrusor overactivity (DO), as defined by the international continence society, is marked by urodynamic observations of uncontrolled contractions of the detrusor muscle during bladder filling, a consequence of cytometric bladder filling or external stimuli such as bending at the waist, while detrusor underactivity (DU) entails contraction of reduced strength and/or duration resulting in prolonged or incomplete emptying of the bladder (Abrams et al. 2003; Foon and Toozs-Hobson 2007; van Koeveringe et al. 2011).

DU is observed in approximately 23% of individuals who undergo urodynamic evaluation for non-neurogenic LUTS, yet the overall prevalence of DU in the general population is obfuscated by the requirement for invasive urodynamic evaluation and ambiguity in clinical and literature definitions (Hartigan et al. 2019; Hoag and Gani 2015; Jeong et al. 2012; Osman et al. 2014). There are no curative treatments for DU, and treatment involves catheterization to aid in bladder emptying (Hartigan et al. 2019). Research on therapeutics for DU may one day include novel drugs, electrical stimulation, and stem cell therapy (Wang et al. 2023).

DO is estimated to occur in approximately 10% of the general population, with increasing prevalence with age with observed occurrence in up to 80% of elderly individuals who undergo urodynamic evaluation for urinary incontinence (Foon and Toozs-Hobson 2007). DO is associated with LUTS including frequency, nocturia, urgency, and urinary incontinence, and the molecular underpinnings of DO are generally understood to have neurogenic and/or myogenic bases (Foon and Toozs-Hobson 2007). In patients with DO coincident with benign prostate hyperplasia and several animal models of DO induced through stricture of the urethra causing partial bladder outlet obstruction, studies observe decreased outward BK channel currents at rest and following detrusor contraction, decreased BKα mRNA and protein expression, and changes to β1 and β4 expression (Aydin et al. 2012; Chang et al. 2010; Hristov et al. 2013; Jiang et al. 2005; Kita et al. 2010; Li et al. 2008; Zheng et al. 2020). Whether the changes to BK channel expression and activity are causal or compensatory in DO remain unclear, however it is likely not a beneficial change considering BK channel’s role in diminishing bladder contractility as discussed in a later section.

Although overactivity and underactivity imply the two conditions are opposites, this can be a misnomer because DO and DU can co-occur, with DO occurring during the filling phase and DU occurring during the voiding phase. DO-DU coincidence occurs in 18.8% and 5.5% of the elderly population of men and women, respectively (Jeong et al. 2012). The two dysfunctions seem to occur independently in men, where 46.5% of men with DU also had DO or BOO and 18.8% of elderly men had detrusor activity co-occurring with impaired bladder contractility (Jeong et al. 2012, 2021). In women, DO-DU may be an intermediate step in the process of DO to DU, where 5.5% of elderly women had detrusor overactivity with impaired contractility (Jeong et al. 2012, 2021).

### Incontinence

Involuntary urinary leakage accompanied by urgent desire to urinate is defined as urgency incontinence (Abrams et al. 2003). OAB without urgency incontinence is termed “OAB dry,” and OAB with urgency incontinence is “OAB wet” which is associated with DO (Corcos and Przydacz 2018; Foon and Toozs-Hobson 2007). In women, the prevalence of OAB dry and OAB wet are similar, while in men OAB dry is more prevalent (Reynolds et al. 2016; Stewart et al. 2003).

Stress urinary incontinence (SUI) is defined as involuntary leakage of urine due to abdominal pressure. Two conditions primarily present in patients presenting with SUI are loss of proximal urethra support due to weakened surrounding muscles leading to a lack of structural support for the urethra, and intrinsic sphincter deficiency causing low-pressure urethra closure (Kayigil et al. 1999; Norton and Brubaker 2006). While these conditions present in both men and women, they are much more common in women due to female-specific traumas to the body, such as vaginal childbirth and hormonal changes in menopause (Norton et al. 1996; Cannon et al. 2003). However, there is growing evidence to suggest that SUI is not only anatomical but may also involve functional deficiencies in the urethral sphincter mechanism (Norton and Brubaker 2006; Kayigil et al. 1999. In adults with neurogenic bladder disease, urethral internal sphincter dysfunction can occur as an additional effect of spinal cord injury, where the urethra contracts and relaxes in accordance with the bladder instead of oppositely (Myers et al. 2016). Furthermore, denervated rat urethras show significant improvement in muscle contraction following treatment with allogenic muscle-derived progenitor cells (Cannon et al. 2003). This underscores the importance of investigating the roles of BK channels and RyR signaling in models of incontinence.

## Urethral obstruction and benign prostatic hyperplasia

The most frequently diagnosed cause of obstruction in males is benign prostatic hyperplasia (BPH), a nonmalignant growth of the prostate that can impede the normal flow of urine from the bladder through the urethra (Ng et al. 2025). BPH is prevalent in older men, with histological evidence observed in approximately 50–60% of men in their 60 s and 80–90% of men over 70 years old (Roehrborn 2005). Evidence has shown that BK channel signaling may play a role in onset and modulation of BPH. Patients with BPH and detrusor overactivity exhibit downregulation of BK channel subunits, suggesting that BK channel dysregulation may contribute to detrusor overactivity symptoms seen in men with BPH (Niwa et al. 2012; Chang et al. 2010).

## BK-RyR coupling in lower urinary tract function

### BK channels

Large-conductance (100–300 pS) voltage- and Ca^2+^-activated K^+^ channels (BK, BK_Ca_, Maxi-K, *Slo1*, or K_Ca_1.1) are potassium ion channels that regulate smooth muscle cell membrane potential through the efflux of potassium ions, and they are the sole K^+^ channel member that is gated by both intracellular Ca^2+^ and membrane voltage. BK channels are expressed in diverse tissues and cell types throughout the body including neurons of the central nervous system (Knaus et al. 1996; Misonou et al. 2006; Sausbier et al. 2006), skeletal muscle cells (Dinardo et al. 2012), and epithelial cells (Cornelius et al. 2020). Most importantly for this review, BK channels are expressed in smooth muscle cells of the bladder (Heppner et al. 1997; Herrera et al. 2000, 2001), urethra (Hollywood et al. 2000; Kyle et al. 2013), uterus (Khan et al. 1997; Wakle-Prabagaran et al. 2016), blood vessels (Dopico et al. 2018; Langton et al. 1991), and airways (Cox and Petrou 1999; Resnik et al. 2006).

The BK channel provides negative feedback for detrusor and urethral contractions, both myogenic and nerve-evoked (Kyle 2014; Kyle et al. 2013; Meredith et al. 2004). Electrophysical studies of bladder and urethra smooth muscle reveal that while other large and small conductance K^+^ channels contribute to depolarization, repolarization and hyperpolarization, the BK channel is the predominant channel involved in maintenance of the resting membrane potential and shaping the current rectification of the repolarization phase (Kyle 2014; Kyle et al. 2013; Meredith et al. 2004). BK channel opening activity can be categorized as either transient or steady state, with the former activated through Ca^2+^ release from the SR/RyR (Ca^2+^ sparks) and the latter activated through Ca^2+^ influx through VDCCs (Collier et al. 2000; Heppner et al. 1997; Herrera and Nelson 2002) (Fig. 1). Thus the BK channel is recognized as a key player in bladder and urethral smooth muscle contraction, and arguably the most important ion channel involved in detrusor contraction (Hayase et al. 2009; Heppner et al. 1997; Kyle 2014; Lee et al. 2018).

BK channels are located within the myocyte plasma membrane, comprised of four alpha (α) subunits as a homotetramer, and when open allows for K^+^ efflux from the myocyte, leading to membrane hyperpolarization and decreased excitability. Changes to the opening probability of the BK channel modulates smooth muscle cell membrane excitability and intracellular Ca^2+^ homeostasis. Increasing intracellular Ca^2+^ concentration and membrane voltage potential both increase the opening probability of the BK channel, but this occurs allosterically with a weak interaction between the two sensors (Gonzalez-Perez and Lingle 2019; Horrigan and Aldrich 2002). Changes to BK channel opening probability are achieved by pre- and post-translational changes to the BK channel, such as phosphorylation by PKA which may be triggered indirectly by M_3_/IP_3_ signaling cascades (Hristov et al. 2014; Parajuli et al. 2015; Parajuli and Petkov 2013). The BK channel is also modified through auxiliary association with beta regulatory subunits, and the less understood gamma regulatory subunits.

Up to four beta subunits (β1-β4) can associate with each BK channel tetramer, with one β subunit binding to each alpha subunit. Increased β subunit association generally sensitizes BK channel opening kinetics when at elevated Ca^2+^ levels and slows closing kinetics, thus modulating channel gating properties (Brenner et al. 2000; Gonzalez-Perez and Lingle 2019; Torres et al. 2014). In the bladder, β1 and β4 are the most abundantly expressed regulatory subunits (Chen and Petkov 2009; Hristov et al. 2011; Jiang et al. 1999; Torres et al. 2014). Increased β1 subunit association with the BK channel increases Ca^2+^ sensitivity, decreases voltage sensitivity, prolongs opening bursts, and slows BK channel closing, while β4 exhibits a biphasic effect on Ca^2+^ sensitivity, reduces voltage sensitivity, and also slows BK channel closing kinetics (Brenner et al. 2000; Contreras et al. 2012; Gonzalez-Perez and Lingle 2019; Ha et al. 2004; Li and Yan 2016; Wang et al. 2006). The presence or absence of β subunits can also impact BK channel sensitivity to pharmacological targeting, with β4 conferring resistance to BK channel blocker, iberiotoxin (Meera et al. 2000).

### Ryanodine receptors

Ryanodine-sensitive Ca^2+^ release channels known as ryanodine receptors (RyRs), are homotetrameric, ligand-gated ion channels predominantly expressed in striated and smooth muscle tissues (Chu et al. 1990; Imagawa et al. 1987; Neylon et al. 1995; Otsu et al. 1990) as well as in neurons (Giannini et al. 1995; Hakamata et al. 1992). Within the cell, RyRs are embedded in the membranes of the sarcoplasmic reticulum (SR)/endoplasmic reticula (ER). Cytoplasmic Ca^2+^ binds to RyRs, inducing a “primed” state with a biphasic sensitivity to Ca^2+^ peaking at approximately 10 to 100 μm [Ca^2+^] (Bezprozvanny et al. 1991). Then ligands (i.e. ATP) bind to and open RyRs to allow Ca^2+^ release from the SR/ER into the cytoplasm (des Georges et al. 2016; Lai et al. 1989; Serysheva 2004). Ryanodine binds to RyRs at nanomolar concentrations, locking the channel in an open state, whereas at higher concentrations (greater than 100 μm), it inhibits Ca^2+^ release (Meissner 1986). Each of the three mammalian RyR isoforms (RyR1, RyR2, Ryr3) are detectable in the urinary bladder, and both RyR1 and RyR2 influence the excitation-contraction coupling of the bladder through interactions with the BK channel (Chambers et al. 1999; Fritz et al. 2007; Hotta et al. 2007; Ji et al. 2004).

### BK-RyR coupling

As illustrated in Fig. 1, in the bladder, nerve-evoked action potentials trigger release of neurotransmitters (acetylcholine and ATP) which activate muscarinic and purinergic receptors on detrusor smooth muscle. This receptor activation leads to membrane depolarization and Ca^2+^ influx through VDCCs triggering contraction (Herrera et al. 2001; Ohi et al. 2001).

BK channels act as a negative-feedback mechanism to regulate detrusor contraction by promoting membrane hyperpolarization and reducing intracellular calcium levels (Herrera et al. 2001; Ohi et al. 2001). BK channels are activated by increases in intracellular Ca^2+^ and/or membrane depolarization. Once open, they allow for the efflux of K+, which results in membrane hyperpolarization. This hyperpolarization reduces Ca^2+^ influx through L-type VDCCs, and decreases excitability (Herrera et al. 2001; Ohi et al. 2001). The functional coupling of BK channels with RyRs plays a key role in regulating bladder micromotions, maintaining tone during bladder filling, and modulating sensitivity to nerve evoked contractile stimuli (Hashitani and Brading 2003; Herrera et al. 2000, 2001; Meredith et al. 2004; Sprossmann et al. 2009; Thorneloe et al. 2005). In the urethra, BK-RyR coupling is similarly involved in maintaining basal tone during bladder filling (Drumm et al. 2018; Feng et al. 2019; Kyle et al. 2013).

RyRs embedded in SR fragments localize near BK channels in detrusor smooth muscle cells (Ohi et al. 2001). While the regulation of BK-RyR colocalization is less studied in myocytes of the lower urinary tract, findings in other tissues support subunit specific interaction, for example β4 subunits promote colocalization of RyR with BK channels in hippocampal neurons of mice (Wang et al. 2016), and pregnancy in sheep increases colocalization of RyR1 and RyR2 with β1 subunits in vascular smooth muscle of uterine arteries (Song et al. 2021). In the bladder, mechanical stretch induces RyR opening and subsequent Ca^2+^ release from the sarcoplasmic reticulum (Ji et al. 2002). These transient Ca^2+^ events contribute to bladder micromotions that trigger afferent nerve activity, facilitating mechanosensation of bladder filling (Chakrabarty et al. 2019; Drake et al. 2017; Heppner et al. 2016; Tran et al. 2025). The physiological relevance of BK-RyR coupling in maintenance of bladder function is further supported by genetic mouse models. BK channel knockout mice exhibit increased strength and frequency of spontaneous detrusor contractions, and increases in dysfunctional urinary voiding including urine leakage (Herrera et al. 2000, 2001; Jiang et al. 1999; Meredith et al. 2004; Thorneloe et al. 2005). In global RyR1 knockout mice, RyR2 and RyR3 could not compensate for the loss of RyR1 in generating depolarization-induced Ca^2+^ sparks in the detrusor (Fritz et al. 2007). Global RyR2 knockout in mice is embryonic lethal; however, mice with heterozygous RyR2 knockdown are viable and detrusor cells display decreased number of Ca^2+^ spark hot spots when depolarized via voltage clamp (< 20 ms), decreased force development in response to electrical stimulation or direct application of acetylcholine, and decreased BK channel opening frequency, indicating that RyR2 is essential for proper BK channel activation and contractile function (Hotta et al. 2007).

### Environmental impacts on BK-RyR coupling and future opportunities

While the etiology of LUTS is complex, there is growing evidence that environmental factors can influence the lower urinary tract and pathophysiology. Below we briefly explore some of these topics and how BK channels and/or RyR signaling pathways could be unexplored areas of considerable interest in improving our understanding of LUTS onset, severity and treatment options.

#### Halogenated biphenyls and Diphenyl ethers activate RyRs and disrupt intracellular calcium dynamics

RyR signaling disruption is correlated with a wide range of disease pathologies, particularly in cardiac muscle, skeletal muscle, and the nervous system (Kushnir et al. 2018; Lanner 2012; Sun et al. 2021). With the increased interest in RyR disruption in the etiology of LUTS, two classes of environmental chemicals of concern are polychlorinated biphenyls (PCBs) and polybrominated diphenyl ethers (PBDEs). Both PCBs and PBDEs are classes of organo-halogenated compounds that have been widely produced and used for industrial and commercial applications, are resistant to environmental degradation, and bioaccumulate in the food chain (Siddiqi et al. 2003). PCBs have been banned for intentional production in the European Union and the USA, however contemporary PCBs are still produced as unintentional byproducts from several industrial processes (Anh et al. 2021). PBDEs have been banned for intentional production EU but remain partially regulated in the USA. Thus PCBs and PBDEs remain a health concern and alarmingly, the PCBs and PBDEs most detected in human samples are also among the most RyR-active (Chen et al. 2017; Kim et al. 2011; Li et al. 2022; Linares et al. 2015; Mouat et al. 2023; Sethi et al. 2019). PCBs and PBDEs are lipophilic and can passively cross plasma membranes, once in the cytoplasm they can bind to RyRs causing abnormal calcium release (Chen et al. 2017; Gong et al. 2021; Kim et al. 2011; Wayman et al. 2012).

In neurons, RyR regulation of intracellular calcium dynamics is important for controlling neuronal outgrowth and electrical firing. Both PCBs and PBDEs bind to RyRs and disrupt RyR homeostatic activity (Coburn et al. 2008; Sethi et al. 2021). Yet, while PCBs increase dendritic arborization, increase axonal complexity, and disrupt intracellular calcium dynamics (Feng et al. 2017; Griffin et al. 2024; Lein 2023; Lesiak et al. 2014; Sethi et al. 2018, 2021, p. 11; Wayman et al. 2012; Wong et al. 1997), PBDEs delay neuronal polarization and axonal outgrowth (Chen et al. 2017; Kim et al. 2011). This difference in PCB and PBDE effects may be due to PCB effects occurring primarily through RyR, while PBDEs also act on IP_3_Rs (Gassmann et al. 2014). Perinatal exposure to PCBs and PBDEs are both associated with neurodevelopmental disorders in rodent models (Kozlova et al. 2022; Li et al. 2021; Sethi et al. 2021) and in longitudinal human studies (Herbstman et al. 2010; Hertz-Picciotto et al. 2018; Lyall et al. 2017; Panesar et al. 2020; Vuong et al. 2018). Neurodevelopmental disorders, including autism spectrum disorder, increase the risk of developing lower urinary tract symptoms, yet the mechanism for how this occurs in children or adults has yet to be determined (Gubbiotti et al. 2019a, b, 2024).

In mouse models, developmental exposure to PCBs results in detectable levels of PCBs in offspring bladder (Keil Stietz et al. 2021), these offspring also display age-, sex-, and dose-dependent changes to urinary voiding physiology, suburothelial nerve density, and sensitivity to intravesical instillation of capsaicin (Keil Stietz et al. 2021; Kennedy et al. 2022, 2021; Lavery et al. 2023; Ridlon et al. 2025). In addition, adult female mice given a human relevant mixture of PCBs during gestation and lactation have increased bladder contractility in response to electrical field stimulation ex vivo (Lavery et al. 2023). These studies highlight the ability of these chemicals to alter the bladder and contractility. Whether this occurs via impacts on bladder or urethra RyR and/or BK channel activity is not currently known but plausible and whether other chemicals like PBDEs could target these pathways to alter voiding function is an ongoing area of study.

#### Endocrine disruption and BK channel activity

Intentional and unintentional changes to endocrine homeostasis are of interest for their impact on LUTS. Hormone therapy is provided to individuals for a variety of treatments including precocious puberty, peri- and post-menopause, birth control, hypogonadism, hair loss, and prostate enlargement (BPH) (Brabaharan et al. 2022; Jarvis et al. 2015; Oesterling 1994; Yang et al. 2025). Conditions necessitating hormone treatments themselves can be risk factors for LUTS, but if hormone treatment itself impacts LUTS is an ongoing area of study (Al-Zoubi et al. 2022; Baas and Köhler 2016; Baruch et al. 2023; Bianchi et al. 2021; Kathrins et al. 2016; Yaish et al. 2025). In mice, treatment with testosterone and estradiol to mimic the hormonal changes of aging men, results in enlarged prostate and an obstruction phenotype (Nicholson et al. 2012). Voiding physiology changes are observed in male mice after undergoing castration or treatment with finasteride (to reduce circulating testosterone levels) or when given exogenous testosterone (Ruetten et al. 2019). Similarly, treatment with testosterone in female mice leads to a more masculine voiding phenotype, and administration of finasteride also impacts voiding even in female mice (Ruetten et al. 2019). Evidence also suggests that BK channels are important for mediating effects of testosterone on the lower urinary tract. Testosterone decreases guinea pig detrusor excitability via increased BK channel open probability and BK channels are necessary for mediating effects of testosterone on detrusor smooth muscle resting potential (Hristov et al. 2016). In vascular smooth muscle, testosterone causes vasodilation in part through activation of BK channels (Lorigo et al. 2020). Estrogens are also important in lower urinary tract function and disease. Estrogen deficiency during and following menopause can lead to urogenital atrophy increasing the risk of urgency urinary incontinence and urinary tract infection (Fait 2019; Henn 2010; Su and Freeman 2009). A recent study from the RISE FOR HEALTH study, conducted by the Prevention of Lower Urinary Tract Symptoms (PLUS) Research Consortium, demonstrates the transition from pre- to perimenopause increases the risk for urgency urinary incontinence, and overall bladder health is worsened in postmenopausal women when undergoing hormone treatment (Vaughan et al. 2025). While this study included a variety of hormone therapies including vaginal estrogen and systemic menopausal hormone therapy, a meta-review of 17 studies reported vulvovaginal topical estrogen therapy improves LUTS in postmenopausal women (Porcari et al. 2025). Ovariectomy in mice, to reduce estrogen levels, leads to mice which display an overactive voiding phenotype which is reversed by estrogen supplementation (Zhang et al. 2024). BK channels are also linked to the actions of estrogens. 17β-Estradiol decreases human bladder smooth muscle cell excitability in part by activating BK channels (Hristov et al. 2017). Estradiol also directly activates BK channels to reduce guinea pig smooth muscle excitability (Provence et al. 2015). 17β-Estradiol directly binds to BK channels in a β-subunit specific manner which increases the channel opening probability primarily in low Ca^2+^ conditions (Behrens et al. 2000; De Wet et al. 2006; Granados et al. 2019; King et al. 2006; Valverde et al. 1999). Additionally, 17β-Estradiol increases BK channel subunit mRNA and/or protein abundance in vascular smooth muscle of sheep (Hu et al. 2011; Nagar et al. 2005) and in neuronal cell lines of mice and humans (Li and Qiu 2015; Nishimura et al. 2008). Using selective agonist and receptor knockdown strategies, these studies confirmed that ERβ mediates the estrogenic regulation of BK channel expression (Li and Qiu 2015; Nishimura et al. 2008).

Environmental chemicals can disrupt the endocrine system broadly by impacting steroidogenesis, mimicking steroid hormones, or altering expression of hormone receptors (Evans 2022). Disruption of steroidogenesis is observed with a wide variety of xenobiotics including PCBs, PBDEs, TCDD (2, 3, 7, 8-tetrachlorodibenzo-*p*-dioxin), and several families of fungicides (Sanderson 2006; Whitehead and Rice 2006). Estrogenic xenobiotics are prevalent environmental contaminants of concern, but the effects of estrogen receptor (ER) activation on BK channel expression/function have not been previously studied in the bladder or urethra of any organism. Environmental estrogen mimicking chemicals including bisphenol A (BPA) and some PCBs can bind to ERs and trigger transcriptional activity (Kuiper et al. 1997). Like estrogens, BPA can bind to BK channels and increase opening probability, but unlike estrogens, the binding site for BPA is on an extracellular site of the α-subunit and sensitivity to BPA is increased by the presence of β1-subunits (Rottgen et al. 2014). BPA has also been shown to increase BK channel expression in rat vascular smooth muscle cells (Costa et al., 2025) and decrease BK channel expression in pancreatic beta cells via ERβ (Martinez-Pinna et al. 2019). Whether BPA or PCBs can similarly alter BK channel expression in the bladder, whether this occurs via Erβ and whether this contributes to voiding dysfunction is currently unknown and is an important area for future research. Further, studies have found that the type of beta subunits present in BK channels can alter the response to steroid hormones (King et al. 2006). Whether this also applies to environmental chemicals is unexplored but potentially impactful as it could add another layer of complexity to understanding how individual responses to environmental chemicals are manifested.Together, these findings underscore the need for further research into how environmental exposures-through modulation of BK and RyR signaling – may contribute to lower urinary tract dysfunction, and highlight these pathways as promising yet underexplored targets for preventive or therapeutic interventions.

## LUTS treatment

Pharmacological interventions for LUTS aimed at controlling detrusor activity are limited to targeting three classes of receptors: muscarinic receptors, the β3 adrenoceptors, or the α1 adrenoceptors (Abreu-Mendes et al. 2020). Non-pharmacologic interventions include pelvic floor exercises and neuromodulation for OAB and stress or mixed incontinence (Yamanishi et al. 2015). In cases of benign prostatic hyperplasia (BPH) associated with LUTS (BPH/LUTS), α1- blockers and β3-agonists, 5α-reductase inhibitors, phosphodiesterase type 5 (PDE5) inhibitors, or a combination drug cocktail may be prescribed (Abreu-Mendes et al. 2020; Laborde and McVary 2009). Antimuscarinic drugs target the muscarinic acetylcholine receptors and improve OAB symptom severity primarily by reducing detrusor smooth muscle contraction (Athanasopoulos and Giannitsas, 2011). While antimuscarinics drugs are generally well tolerated and improve symptom severity, muscarinic receptors are present in tissues outside of the urinary tract, leading to the drugs having side effects including dry mouth, dry eyes, and constipation (Foon and Toozs-Hobson 2007; Madhuvrata et al. 2012).

The next generation of LUTS treatments, especially when related to dysfunction of the smooth muscle of the lower urinary tract, may focus on BK channels and RyRs, either alone or in combination. However, it is important to note that BK channels and RyRs are also widely expressed outside of the lower urinary tract, and their individual and coupled functions are important in regulating bladder and urethra contractile states during different phases of the micturition cycle. Therefore, when developing therapeutics aimed at modulating BK channels or RyRs, caution must be exercised to minimize off target effects across lower urinary tract tissue and throughout the body to ensure a quality of care similar to other available therapeutics. For example, NS-8 (CAS RN 186033-14-7) was in a proof-of-concept clinical trials (EudraCT Number: 2005-001250-24) for overactive bladder treatment (120, 180, or 240 mg twice a day orally for 56 days) in men and women through activation of BK channels and other potassium ion channels; however it was discontinued in phase two due to a lack of efficacy in reducing incontinence episodes (Nardi and Olesen 2008), Likewise, BMS204352 (CAS RN 187523-35-9, Maxipost) was in clinical trials for ischemic stroke treatment (0.3 mg/kg, i.v.) through activation of BK channels and other ion channels; however it was discontinued in phase three due to a lack of efficiency in acute stroke patients (Jensen 2002) and intravenous administration (two doses of 1 mg over 20 min) causes migraines in healthy volunteers (Al-Karagholi et al. 2020, 2021). Given that BK channels and RyRs function in diverse cell types throughout the body, and their roles during bladder filling and emptying, increasing BK channel or RyR opening probability without cell or tissue specific targeting is unlikely to provide a universal solution to LUTS (Sancho and Kyle 2021).

Promising results arose from recent studies targeting BK channels and RyRs separately with greater specificity than previous trials. Recently a potential therapeutic compound, the BK channel opener LDD175, improved erectile dysfunction symptoms and LUTS in rats (Yu et al. 2025). URO-902, a gene therapy recently in clinical trials (NCT04211831), that increases BKα expression targeting smooth muscle cells, reportedly improves symptoms of overactive bladder, urgency urinary incontinence, and erectile dysfunction (Andersson et al. 2021; Enemchukwu et al. 2025; Melman et al. 2006). For treatment of men with Fragile X Syndrome, phase two of clinical trials (NCT06413537) was recently completed using the BK channel opener SPG601 (8 capsules of 100 mg on two separate days). Strategies focused on RyR activity are of interest for treatment of pathologies beyond the lower urinary tract including neurodegenerative diseases such as Alzheimer’s Disease and heart failure due to cardiac arrhythmias (Lacampagne et al. 2017; Liu et al. 2012, p. 201; Sun et al. 2021); However, like environmental disruptors of RyR activity, chronic activation of RyR results in a loss in Ca^2+^ stores and therefore loss of controlled muscle contraction (Andronache et al. 2007; Bellinger et al. 2008). S 48,168 (ARM 210) was recently in phase 1 clinical trials (NCT04141670) for treatment of skeletal myopathies caused by pathogenic variants in the *RYR1* genes leading to Ca^2+^ leak from the SR, with treatment (120 mg or 200 mg daily for 29 days) showing promising results to proceed to further trials (Todd et al. 2024).

Therefore, although BK channels or RyRs pose an exciting opportunity for novel therapeutics due to their central role in smooth muscle contractility, therapies must be developed with specificity and caution to avoid systemic side effects. Nonetheless, as we continue to understand more about the biology of these channels, particularly in relation to risk factors such as environmental contaminants that may disrupt their function, there is significant potential for developing targeted therapies for individuals with these pathways disrupted or with significant environmental exposures which could provide improved personalized and effective treatment options.

## Data Availability

No datasets were generated or analysed during the current study.

## References

[CR1] Abelson B, Sun D, Que L, Nebel RA, Baker D, Popiel P, Amundsen CL, Chai T, Close C, DiSanto M, Fraser MO, Kielb SJ, Kuchel G, Mueller ER, Palmer MH, Parker-Autry C, Wolfe AJ, Damaser MS (2018) Sex differences in lower urinary tract biology and physiology. Biol Sex Differ 9:45. 10.1186/s13293-018-0204-8 30343668 10.1186/s13293-018-0204-8 PMC6196569

[CR2] Abrams P, Cardozo L, Fall M, Griffiths D, Rosier P, Ulmsten U, Van Kerrebroeck P, Victor A, Wein A, Standardisation Sub-Committee of the International Continence Society (2003) The standardisation of terminology in lower urinary tract function: report from the standardisation sub-committee of the international continence society. Urology 61:37–49. 10.1016/s0090-4295(02)02243-4 12559262 10.1016/s0090-4295(02)02243-4

[CR3] Abreu-Mendes P, Silva J, Cruz F (2020) Pharmacology of the lower urinary tract: update on LUTS treatment. Ther Adv Urol 12:1756287220922425. 10.1177/1756287220922425 32489425 10.1177/1756287220922425 PMC7238773

[CR4] Aggarwal H, Zimmern PE (2016) Underactive bladder. Curr Urol Rep 17:1–10. 10.1007/s11934-016-0582-6 26874529 10.1007/s11934-016-0582-6

[CR5] Al-Karagholi MA-M, Ghanizada H, Nielsen CAW, Skandarioon C, Snellman J, Lopez Lopez C, Hansen JM, Ashina M (2020) Opening of BKCa channels alters cerebral hemodynamic and causes headache in healthy volunteers. Cephalalgia Int J Headache 40:1145–1154. 10.1177/0333102420940681 10.1177/0333102420940681 32847403

[CR6] Al-Karagholi MA-M, Ghanizada H, Nielsen W, Skandarioon CA, Snellman C, Lopez-Lopez J, Hansen C, Ashina JM, M (2021) Opening of BKCa channels causes migraine attacks: a new downstream target for the treatment of migraine. Pain 162:2512–2520. 10.1097/j.pain.0000000000002238 34252916 10.1097/j.pain.0000000000002238

[CR7] Al-Zoubi RM, Alwani M, Aboumarzouk OM, Elaarag M, Al-Qudimat AR, Ojha L, Yassin A (2022) Updates on androgen replacement therapy and lower urinary tract symptoms: a narrative review. Aging Male 25:234–241. 10.1080/13685538.2022.2118253 36066424 10.1080/13685538.2022.2118253

[CR8] Ambrogi M, Hernandez LL, Strand DW, Kumar S, Romero MF, Barasch J, Ridlon M, Keil Stietz KP, Vezina CM (2025) A 5-HT-mediated urethral defense against urinary tract infections. Proc Natl Acad Sci U S A 122:e2409754122. 10.1073/pnas.2409754122 40228121 10.1073/pnas.2409754122 PMC12037003

[CR9] Andersson K-E, McCloskey KD (2014) Lamina propria: the functional center of the bladder? Neurourol Urodyn 33:9–16. 10.1002/nau.22465 23847015 10.1002/nau.22465

[CR10] Andersson KE, Garcia Pascual A, Forman A, Tøttrup A (1991) Non-adrenergic, non-cholinergic nerve-mediated relaxation of rabbit urethra is caused by nitric oxide. Acta Physiol Scand 141:133–134. 10.1111/j.1748-1716.1991.tb09056.x 2053441 10.1111/j.1748-1716.1991.tb09056.x

[CR11] Andersson K-E, Pascual AG, Persson K, Forman A, Tøttrup A (1992) Electrically-induced, nerve-mediated relaxation of rabbit urethra involves nitric oxide. J Urol 147:253–259. 10.1016/S0022-5347(17)37208-7 1729542 10.1016/s0022-5347(17)37208-7

[CR12] Andersson K-E, Christ GJ, Davies KP, Rovner ES, Melman A (2021) Gene therapy for overactive bladder: a review of BK-channel α-subunit gene transfer. Ther Clin Risk Manag 17:589–599. 10.2147/TCRM.S291798 34113116 10.2147/TCRM.S291798 PMC8187094

[CR13] Andronache Z, Ursu D, Lehnert S, Freichel M, Flockerzi V, Melzer W (2007) The auxiliary subunit γ1 of the skeletal muscle L-type Ca2 + channel is an endogenous Ca2 + antagonist. Proc Natl Acad Sci U S A 104:17885–17890. 10.1073/pnas.0704340104 17978188 10.1073/pnas.0704340104 PMC2077065

[CR14] Anh HQ, Watanabe I, Minh TB, Takahashi S (2021) Unintentionally produced polychlorinated biphenyls in pigments: an updated review on their formation, emission sources, contamination status, and toxic effects. Sci Total Environ 755:142504. 10.1016/j.scitotenv.2020.142504 33035974 10.1016/j.scitotenv.2020.142504

[CR15] Aydin M, Wang HZ, Zhang X, Chua R, Downing K, Melman A, DiSanto ME (2012) Large-conductance calcium-activated potassium channel activity, as determined by whole-cell patch clamp recording, is decreased in urinary bladder smooth muscle cells from male rats with partial urethral obstruction. BJU Int 110:E402–E408. 10.1111/j.1464-410X.2012.11137.x 22520450 10.1111/j.1464-410X.2012.11137.x

[CR16] Baas W, Köhler TS (2016) Testosterone replacement therapy and BPH/LUTS. What is the evidence?? Curr. Urol Rep 17:1–5. 10.1007/s11934-016-0600-8 10.1007/s11934-016-0600-8 27068735

[CR17] Baruch Y, Torella M, De Bastiani S, Meschia M, Candiani M, Colacurci N, Salvatore S (2023) Pre- versus Post-Menopausal onset of overactive bladder and the response to vaginal Estrogen therapy: A prospective study. Med (Mex 59:245. 10.3390/medicina59020245 10.3390/medicina59020245 PMC9963172 36837446

[CR18] Behrens R, Nolting A, Reimann F, Schwarz M, Waldschütz R, Pongs O (2000) Hkcnmb3 and hkcnmb4, cloning and characterization of two members of the large-conductance calcium-activated potassium channel β subunit family. FEBS Lett 474:99–106. 10.1016/S0014-5793(00)01584-2 10828459 10.1016/s0014-5793(00)01584-2

[CR19] Bellinger AM, Reiken S, Dura M, Murphy PW, Deng S-X, Landry DW, Nieman D, Lehnart SE, Samaru M, LaCampagne A, Marks AR (2008) Remodeling of ryanodine receptor complex causes leaky channels: a molecular mechanism for decreased exercise capacity. Proc Natl Acad Sci U S A 105:2198–2202. 10.1073/pnas.0711074105 18268335 10.1073/pnas.0711074105 PMC2538898

[CR20] Bezprozvanny L, Watras J, Ehrlich BE (1991) Bell-shaped calcium-response curves of lns(l,4,5)P3- and calcium-gated channels from endoplasmic reticulum of cerebellum. Nature 351:751–754. 10.1038/351751a0 1648178 10.1038/351751a0

[CR21] Bharti V, Tiwari RK, Gupta S, Upadhyay R, Singh MK, Singh DK (2023) The spectrum and etiologies of lower urinary tract symptoms in postmenopausal women. Curr Urol 17:179–183. 10.1097/CU9.0000000000000196 37448608 10.1097/CU9.0000000000000196 PMC10337809

[CR22] Bianchi VE, Bresciani E, Meanti R, Rizzi L, Omeljaniuk RJ, Torsello A (2021) The role of androgens in women’s health and wellbeing. Pharmacol Res 171:105758. 10.1016/j.phrs.2021.105758 34242799 10.1016/j.phrs.2021.105758

[CR23] Bolla SR, Odeluga N, Amraei R, Jetti R (2025) Histology, Bladder. StatPearls. StatPearls Publishing, Treasure Island (FL) 31082007

[CR24] Brabaharan S, Veettil SK, Kaiser JE, Raja Rao VR, Wattanayingcharoenchai R, Maharajan M, Insin P, Talungchit P, Anothaisintawee T, Thakkinstian A, Chaiyakunapruk N (2022) Association of hormonal contraceptive use with adverse health outcomes: an umbrella review of meta-analyses of randomized clinical trials and cohort studies. JAMA Netw Open 5:e2143730. 10.1001/jamanetworkopen.2021.43730 35029663 10.1001/jamanetworkopen.2021.43730 PMC8760614

[CR243] Brading AF, Teramoto T, Nakayama S, Bramich N, Inoue R, Fujii K, Mostwin J (1996) The relationship between the electrophysiological properties of lower urinary tract smooth muscles and their function in vivo. In: Bolton TB, Tonita T, editors. Smooth Muscle Excitation. London: Academic Press; pp. 403–415.

[CR25] Brading AF (1997) A myogenic basis for the overactive bladder. Urology 50:57–67. 10.1016/S0090-4295(97)00591-8 9426752 10.1016/s0090-4295(97)00591-8

[CR26] Brading AF (1999) The physiology of the mammalian urinary outflow tract. Exp Physiol 84:215–221. 10.1111/j.1469-445x.1999.tb00084.x 10081719 10.1111/j.1469-445x.1999.tb00084.x

[CR27] Brading AF (2002) The Sarcoplasmic Reticulum in Disease and Smooth muscle Dysfunction: Therapeutic Potential, in: Role Of The Sarcoplasmic Reticulum. Smooth muscle. John Wiley & Sons, Ltd, pp 244–257. 10.1002/0470853050.ch18 10.1002/0470853050.ch18 12164312

[CR28] Bradley E, Kadima S, Kyle B, Hollywood MA, Thornbury KD, McHale NG, Sergeant GP (2011) P2X receptor currents in smooth muscle cells contribute to nerve mediated contractions of rabbit urethral smooth muscle. J Urol 186:745–752. 10.1016/j.juro.2011.03.140 21683405 10.1016/j.juro.2011.03.140 PMC5793217

[CR29] Brenner R, Jegla TJ, Wickenden A, Liu Y, Aldrich RW (2000) Cloning and functional characterization of novel large conductance calcium-activated potassium channel β subunits, hKCNMB3 and hKCNMB4. J Biol Chem 275:6453–6461. 10.1074/jbc.275.9.6453 10692449 10.1074/jbc.275.9.6453

[CR30] Burnstock G (2014) Purinergic signalling: from discovery to current developments. Exp Physiol 99:16–34. 10.1113/expphysiol.2013.071951 24078669 10.1113/expphysiol.2013.071951 PMC4208685

[CR31] Callahan SM, Creed KE (1981) Electrical and mechanical activity of the isolated lower urinary tract of the guinea-pig. Br J Pharmacol 74:353–358. 10.1111/j.1476-5381.1981.tb09978.x 7198498 10.1111/j.1476-5381.1981.tb09978.x PMC2071739

[CR32] Cannon TW, Lee JY, Somogyi G, Pruchnic R, Smith CP, Huard J, Chancellor MB (2003) Improved sphincter contractility after allogenic muscle-derived progenitor cell injection into the denervated rat urethra. Urology 62:958–963. 10.1016/s0090-4295(03)00679-4 14624934 10.1016/s0090-4295(03)00679-4

[CR33] Chakrabarty B, Bijos DA, Vahabi B, Clavica F, Kanai AJ, Pickering AE, Fry CH, Drake MJ (2019) Modulation of bladder wall micromotions alters intravesical pressure activity in the isolated bladder. Front Physiol. 10.3389/fphys.2018.01937 30687132 10.3389/fphys.2018.01937 PMC6335571

[CR34] Chambers P, Neal DE, Gillespie JI (1999) Ryanodine receptors in human bladder smooth muscle. Exp Physiol 84:41–46. 10.1111/j.1469-445X.1999.tb00070.x 10081705 10.1111/j.1469-445x.1999.tb00070.x

[CR35] Chang S, Gomes CM, Hypolite JA, Marx J, Alanzi J, Zderic SA, Malkowicz B, Wein AJ, Chacko S (2010) Detrusor overactivity is associated with downregulation of large-conductance calcium- and voltage-activated potassium channel protein. Am J Physiol-Ren Physiol 298:F1416–F1423. 10.1152/ajprenal.00595.2009 10.1152/ajprenal.00595.2009 PMC2886817 20392804

[CR36] Chapple CR, Yamanishi T, Chess-Williams R (2002) Muscarinic receptor subtypes and management of the overactive bladder. Urology 60:82–88. 10.1016/s0090-4295(02)01803-4. discussion 88–89 12493364 10.1016/s0090-4295(02)01803-4

[CR37] Chen M, Petkov GV (2009) Identification of large conductance calcium activated potassium channel accessory β4 subunit in rat and mouse bladder smooth muscle. J Urol 182:374–381. 10.1016/j.juro.2009.02.109 19447431 10.1016/j.juro.2009.02.109 PMC4461143

[CR38] Chen H, Streifel KM, Singh V, Yang D, Mangini L, Wulff H, Lein PJ (2017) From the cover: BDE-47 and BDE-49 inhibit axonal growth in primary rat hippocampal neuron-glia co-cultures via ryanodine receptor-dependent mechanisms. Toxicol Sci 156:375–386. 10.1093/toxsci/kfw259 28003438 10.1093/toxsci/kfw259 PMC6070026

[CR39] Chess-Williams R (2002) Muscarinic receptors of the urinary bladder: detrusor, urothelial and prejunctional. Auton Autacoid Pharmacol 22:133–145. 10.1046/j.1474-8673.2002.00258.x 12452898 10.1046/j.1474-8673.2002.00258.x

[CR40] Chess-Williams R, Sellers DJ (2023) Pathophysiological mechanisms involved in overactive bladder/detrusor overactivity. Curr Bladder Dysfunct Rep 18:79–88. 10.1007/s11884-023-00690-x

[CR41] Christ GJ, Hodges S (2006) Molecular mechanisms of detrusor and corporal myocyte contraction: identifying targets for pharmacotherapy of bladder and erectile dysfunction. Br J Pharmacol 147:S41–S55. 10.1038/sj.bjp.0706627 16465183 10.1038/sj.bjp.0706627 PMC1751499

[CR42] Chu A, Díaz-Muñoz M, Hawkes MJ, Brush K, Hamilton SL (1990) Ryanodine as a probe for the functional state of the skeletal muscle sarcoplasmic reticulum calcium release channel. Mol Pharmacol 37(5):735–741 1692609

[CR43] Clapham DE (ed) (1996) Organellar ion channels and transporters: 7–9 September 1995 [at the Marine Biological Laboratory in Woods Hole, Massachusetts], Annual symposium / Society of General Physiologists. Rockefeller Univ. Press, New York

[CR44] Coburn CG, Currás-Collazo MC, Kodavanti PRS (2008) In vitro effects of environmentally relevant polybrominated diphenyl ether (PBDE) congeners on calcium buffering mechanisms in rat brain. Neurochem Res 33:355–364. 10.1007/s11064-007-9430-x 17846885 10.1007/s11064-007-9430-x

[CR45] Collier ML, Ji G, Wang Y-X, Kotlikoff MI (2000) Calcium-induced calcium release in smooth muscle: loose coupling between the action potential and calcium release. J Gen Physiol 115:653–662. 10.1085/jgp.115.5.653 10779321 10.1085/jgp.115.5.653 PMC2217224

[CR46] Contreras GF, Neely A, Alvarez O, Gonzalez C, Latorre R (2012) Modulation of BK channel voltage gating by different auxiliary β subunits. Proc Natl Acad Sci 109:18991–18996. 10.1073/pnas.1216953109 23112204 10.1073/pnas.1216953109 PMC3503226

[CR47] Corcos J, Przydacz M (2018) Incontinence due to neurogenic detrusor overactivity. In: Corcos J, Przydacz M (eds) Consultation in neurourology: A practical Evidence-Based guide. Springer International Publishing, Cham, pp 77–113. 10.1007/978-3-319-63910-9_7

[CR48] Cornelius RJ, Wang-France J, Sansom SC (2020) BK channels in epithelia. Studies of epithelial transporters and ion channels. Springer, Cham, pp 949–965. 10.1007/978-3-030-55454-5_23

[CR259] Costa HE, Lorigo M, Cairrao E (2025) Bisphenol A Exposure Modifies the Vasoactive Response of the Middle Cerebral Artery. Int J Mol Sci 26(8):3896. 10.3390/ijms26083896 10.3390/ijms26083896 PMC12027836 40332806

[CR49] Cox RH, Petrou S (1999) Ca2 + influx inhibits voltage-dependent and augments Ca2+-dependent K + currents in arterial myocytes. Am J Physiol-Cell Physiol 277:C51–C63. 10.1152/ajpcell.1999.277.1.C51 10.1152/ajpcell.1999.277.1.C51 10409108

[CR50] Damaser MS, Kim FJ, Minetti GM (2003) Methods of testing urethral resistance in the female rat. Adv Exp Med Biol 539:831–839. 10.1007/978-1-4419-8889-8_51 15176326 10.1007/978-1-4419-8889-8_51

[CR51] Daniel EE, Cowan W, Daniel VP (1983) Structural bases for neural and myogenic control of human detrusor muscle. Can J Physiol Pharmacol 61:1247–1273. 10.1139/y83-183 6661682 10.1139/y83-183

[CR52] De Wet H, Allen M, Holmes C, Stobbart M, Lippiat JD, De Wet H, Allen M, Holmes C, Stobbart M, Lippiat JD, Callaghan R (2006) Modulation of the BK channel by estrogens: examination at single channel level. Mol Membr Biol 23:420–429. 10.1080/09687860600802803 17060159 10.1080/09687860600802803

[CR53] DeLancey JOL, Trowbridge ER, Miller JM, Morgan DM, Guire K, Fenner DE, Weadock WJ, Ashton-Miller JA (2008) Stress urinary incontinence: relative importance of urethral support and urethral closure pressure. J Urol 179:2286–2290. 10.1016/j.juro.2008.01.098 18423707 10.1016/j.juro.2008.01.098 PMC2673985

[CR54] des Georges A, Clarke OB, Zalk R, Yuan Q, Condon KJ, Grassucci RA, Hendrickson WA, Marks AR, Frank J (2016) Structural basis for gating and activation of RyR1. Cell 167:145–157e17. 10.1016/j.cell.2016.08.075 27662087 10.1016/j.cell.2016.08.075 PMC5142848

[CR55] Dinardo MM, Camerino G, Mele A, Latorre R, Conte Camerino D, Tricarico D (2012) Splicing of the rSlo gene affects the molecular composition and drug response of Ca2+-activated K + channels in skeletal muscle. PLoS One 7:e40235. 10.1371/journal.pone.0040235 22808126 10.1371/journal.pone.0040235 PMC3393747

[CR56] Dopico AM, Bukiya AN, Jaggar JH (2018) Calcium- and voltage-gated BK channels in vascular smooth muscle. Pflugers Arch 470:1271–1289. 10.1007/s00424-018-2151-y 29748711 10.1007/s00424-018-2151-y PMC6417838

[CR57] Drake MJ, Kanai A, Bijos DA, Ikeda Y, Zabbarova I, Vahabi B, Fry CH (2017) The potential role of unregulated autonomous bladder micromotions in urinary storage and voiding dysfunction; overactive bladder and detrusor underactivity. BJU Int 119:22–29. 10.1111/bju.13598 27444952 10.1111/bju.13598 PMC5177525

[CR58] Drumm BT, Rembetski BE, Cobine CA, Baker SA, Sergeant GP, Hollywood MA, Thornbury KD, Sanders KM (2018) Ca2 + signalling in mouse urethral smooth muscle in situ: role of Ca2 + stores and Ca2 + influx mechanisms. J Physiol 596:1433–1466. 10.1113/JP275719 29383731 10.1113/JP275719 PMC5899989

[CR59] Drumm BT, Gupta N, Mircea A, Griffin CS (2024) Cells and ionic conductances contributing to spontaneous activity in bladder and urethral smooth muscle. J Physiol. 10.1113/JP284744 39323077 10.1113/JP284744 PMC13134435

[CR60] Eapen RS, Radomski SB (2016) Review of the epidemiology of overactive bladder. Res Rep Urol 8:71–76. 10.2147/RRU.S102441 27350947 10.2147/RRU.S102441 PMC4902138

[CR61] Eggermont M, De Wachter S, Eastham J, Gillespie J (2019) Innervation of the epithelium and lamina propria of the urethra of the female rat. Anat Rec 302(2):201–214. 10.1002/ar.23937 10.1002/ar.23937 30290399

[CR62] Elbadawi A, Yalla SV, Resnick NM (1993) Structural basis of geriatric voiding dysfunction. Part II. Aging detrusor: normal versus impaired contractility. J Urol 150:1657–1667. 10.1016/s0022-5347(17)35867-6 8411454 10.1016/s0022-5347(17)35867-6

[CR63] Enemchukwu EA, Kalota S, Robertson K, Ge S, Lu J, Badger H, Mujais S, Peters KM (2025) Gene therapy with URO-902 (pVAX/hSlo) for the treatment of female patients with overactive bladder and urge urinary incontinence: safety and efficacy from a randomized phase 2a trial. J Urol. 10.1097/JU.0000000000004373 39693268 10.1097/JU.0000000000004373 PMC12708029

[CR64] Evans TJ (2022) Chapter 58 - Endocrine disruption, in: Gupta, R.C. (Ed.), Reproductive and Developmental Toxicology (Third Edition). Academic Press, pp. 1163–1182. 10.1016/B978-0-323-89773-0.00058-8

[CR65] Fait T (2019) Menopause hormone therapy: latest developments and clinical practice. Drugs Context 8:212551. 10.7573/dic.212551 30636965 10.7573/dic.212551 PMC6317580

[CR66] Feng W, Zheng J, Robin G, Dong Y, Ichikawa M, Inoue Y, Mori T, Nakano T, Pessah IN (2017) Enantioselectivity of 2,2′,3,5′,6-pentachlorobiphenyl (PCB 95) atropisomers toward Ryanodine receptors (RyRs) and their influences on hippocampal neuronal networks. Environ Sci Technol 51:14406–14416. 10.1021/acs.est.7b04446 29131945 10.1021/acs.est.7b04446 PMC6251309

[CR67] Feng M, Wang Z, Liu Z, Liu D, Zheng K, Lu P, Liu C, Zhang M, Li J (2019) The RyR-ClCa -VDCC axis contributes to spontaneous tone in urethral smooth muscle. J Cell Physiol 234:23256–23267. 10.1002/jcp.28892 31161632 10.1002/jcp.28892

[CR68] Ferreira A, Duarte Cruz C (2021) The urethra in continence and sensation: neural aspects of urethral function. Neurourol Urodyn 40:744–752. 10.1002/nau.24632 33604909 10.1002/nau.24632

[CR69] Foon R, Toozs-Hobson P (2007) Detrusor overactivity. Obstet Gynaecol Reprod Med 17:255–260. 10.1016/j.ogrm.2007.07.004

[CR70] Fritz N, Morel J-L, Jeyakumar LH, Fleischer S, Allen PD, Mironneau J, Macrez N (2007) RyR1-specific requirement for depolarization-induced Ca2 + sparks in urinary bladder smooth muscle. J Cell Sci 120:3784–3791. 10.1242/jcs.009415 17925380 10.1242/jcs.009415

[CR71] Gassmann K, Schreiber T, Dingemans MML, Krause G, Roderigo C, Giersiefer S, Schuwald J, Moors M, Unfried K, Bergman Å, Westerink RHS, Rose CR, Fritsche E (2014) BDE-47 and 6-OH-BDE-47 modulate calcium homeostasis in primary fetal human neural progenitor cells via ryanodine receptor-independent mechanisms. Arch Toxicol 88:1537–1548. 10.1007/s00204-014-1217-7 24599297 10.1007/s00204-014-1217-7

[CR72] Giannini G, Conti A, Mammarella S, Scrobogna M, Sorrentino V (1995) The ryanodine receptor/calcium channel genes are widely and differentially expressed in murine brain and peripheral tissues. J Cell Biol 128:893–904. 10.1083/jcb.128.5.893 7876312 10.1083/jcb.128.5.893 PMC2120385

[CR73] Giglio D, Tobin G (2009) Muscarinic receptor subtypes in the lower urinary tract. Pharmacology 83:259–269. 10.1159/000209255 19295256 10.1159/000209255

[CR74] Gong D, Yan N, Ledford HA (2021) Structural basis for the modulation of Ryanodine receptors. Trends Biochem Sci 46:489–501. 10.1016/j.tibs.2020.11.009 33353849 10.1016/j.tibs.2020.11.009

[CR75] Gonzalez-Perez V, Lingle CJ (2019) Regulation of BK channels by beta and gamma subunits. Annu Rev Physiol 81(1):113–137. 10.1146/annurev-physiol-022516-034038 30742788 10.1146/annurev-physiol-022516-034038 PMC6380188

[CR76] Gormley EA, Lightner DJ, Faraday M, Vasavada SP (2015) Diagnosis and treatment of overactive bladder (non-neurogenic) in adults: AUA/SUFU guideline amendment. J Urol. 10.1016/j.juro.2015.01.087 25623739 10.1016/j.juro.2015.01.087

[CR77] Granados ST, Castillo K, Bravo-Moraga F, Sepúlveda RV, Carrasquel-Ursulaez W, Rojas M, Carmona E, Lorenzo-Ceballos Y, González-Nilo F, González C, Latorre R, Torres YP (2019) The molecular nature of the 17β-Estradiol binding site in the voltage- and Ca2+-activated K+ (BK) channel β1 subunit. Sci Rep 9:9965. 10.1038/s41598-019-45942-1 31292456 10.1038/s41598-019-45942-1 PMC6620312

[CR78] Greenland JE, Dass N, Brading AF (1996) Intrinsic urethral closure mechanisms in the female pig. Scand J Urol Nephrol Suppl 179:75–80 8908669

[CR79] Griffin JA, Li X, Lehmler H-J, Holland EB (2024) Predicted versus observed activity of PCB mixtures toward the Ryanodine receptor. Neurotoxicology 100:25–34. 10.1016/j.neuro.2023.12.003 38065417 10.1016/j.neuro.2023.12.003 PMC10842331

[CR80] Griffiths D, Tadic SD (2008) Bladder control, urgency, and urge incontinence: evidence from functional brain imaging. Neurourol Urodyn 27:466–474. 10.1002/nau.20549 18092336 10.1002/nau.20549

[CR81] Gubbiotti M, Balboni G, Bini V, Elisei S, Bedetti C, Marchiafava M, Giannantoni A (2019a) Bladder and bowel dysfunction, adaptive behaviour and psychiatric profiles in adults affected by autism spectrum disorders. Neurourol Urodyn 38:1866–1873. 10.1002/nau.24081 31270838 10.1002/nau.24081

[CR82] Gubbiotti M, Elisei S, Bedetti C, Marchiafava M, Giannantoni A (2019b) Urinary and bowel disfunction in autism spectrum disorder: a prospective, observational study. Psychiatr Danub 31:475–478 31488775

[CR83] Gubbiotti M, Balzarro M, Zoccante L, Di Gennaro G, Marchiafava M, Bedetti C, Rubilotta E (2024) National survey on bladder and bowel dysfunctions in autism spectrum disorder population. Front Psychiatry 15:1140113. 10.3389/fpsyt.2024.1140113 38528973 10.3389/fpsyt.2024.1140113 PMC10961907

[CR84] Ha TS, Heo M-S, Park C-S (2004) Functional effects of auxiliary beta4-subunit on rat large-conductance Ca(2+)-activated K(+) channel. Biophys J 86:2871–2882. 10.1016/S0006-3495(04)74339-8 15111404 10.1016/S0006-3495(04)74339-8 PMC1304156

[CR85] Hakamata Y, Nakai J, Takeshima H, Imoto K (1992) Primary structure and distribution of a novel Ryanodine receptor/calcium release channel from rabbit brain. FEBS Lett 312:229–235. 10.1016/0014-5793(92)80941-9 1330694 10.1016/0014-5793(92)80941-9

[CR86] Hartigan SM, Reynolds WS, Dmochowski RR (2019) Detrusor underactivity in women: a current understanding. Neurourol Urodyn 38:2070–2076. 10.1002/nau.24147 31432566 10.1002/nau.24147

[CR87] Hashitani H, Brading AF (2003) Electrical properties of detrusor smooth muscles from the pig and human urinary bladder. Br J Pharmacol 140:146–158. 10.1038/sj.bjp.0705319 12967944 10.1038/sj.bjp.0705319 PMC1573994

[CR88] Hayase M, Hashitani H, Kohri K, Suzuki H (2009) Role of K + channels in regulating spontaneous activity in detrusor smooth muscle in situ in the mouse bladder. J Urol 181:2355–2365. 10.1016/j.juro.2009.01.013 19303094 10.1016/j.juro.2009.01.013

[CR89] Heesakkers JPFA, Gerretsen RRR (2004) Urinary incontinence: sphincter functioning from a urological perspective. Digestion 69:93–101. 10.1159/000077875 15087576 10.1159/000077875

[CR260] Henn E (2010) Menopause and its effect on the female lower urinary tract. South African Fam Pract 52:405–408. 10.1080/20786204.2010.10874015

[CR90] Henry GH, Malewska A, Joseph DB, Malladi VS, Lee J, Torrealba J, Mauck RJ, Gahan JC, Raj GV, Roehrborn CG, Hon GC, MacConmara MP, Reese JC, Hutchinson RC, Vezina CM, Strand DW (2018) A cellular anatomy of the normal adult human prostate and prostatic urethra. Cell Rep 25:3530–3542e5. 10.1016/j.celrep.2018.11.086 30566875 10.1016/j.celrep.2018.11.086 PMC6411034

[CR91] Heppner TJ, Bonev AD, Nelson MT (1997) Ca(2+)-activated K + channels regulate action potential repolarization in urinary bladder smooth muscle. Am J Physiol 273:C110–117. 10.1152/ajpcell.1997.273.1.C110 9252448 10.1152/ajpcell.1997.273.1.C110

[CR92] Heppner TJ, Werner ME, Nausch B, Vial C, Evans RJ, Nelson MT (2009) Nerve-evoked purinergic signalling suppresses action potentials, Ca2 + flashes and contractility evoked by muscarinic receptor activation in mouse urinary bladder smooth muscle. J Physiol 587:5275–5288. 10.1113/jphysiol.2009.178806 19736301 10.1113/jphysiol.2009.178806 PMC2790264

[CR93] Heppner TJ, Tykocki NR, Hill-Eubanks D, Nelson MT (2016) Transient contractions of urinary bladder smooth muscle are drivers of afferent nerve activity during filling. J Gen Physiol 147:323–335. 10.1085/jgp.201511550 26976828 10.1085/jgp.201511550 PMC4810069

[CR94] Herbstman JB, Sjödin A, Kurzon M, Lederman SA, Jones RS, Rauh V, Needham LL, Tang D, Niedzwiecki M, Wang RY, Perera F (2010) Prenatal exposure to PBDEs and neurodevelopment. Environ Health Perspect 118:712–719. 10.1289/ehp.0901340 20056561 10.1289/ehp.0901340 PMC2866690

[CR95] Herrera GM, Nelson MT (2002) Differential regulation of SK and BK channels by Ca2 + signals from Ca2 + channels and ryanodine receptors in guinea-pig urinary bladder myocytes. J Physiol 541:483–492. 10.1113/jphysiol.2002.017707 12042353 10.1113/jphysiol.2002.017707 PMC2290319

[CR96] Herrera GM, Heppner TJ, Nelson MT (2000) Regulation of urinary bladder smooth muscle contractions by ryanodine receptors and BK and SK channels. Am J Physiol-Regul Integr Comp Physiol 279:R60–R68. 10.1152/ajpregu.2000.279.1.R60 10896865 10.1152/ajpregu.2000.279.1.R60

[CR97] Herrera GM, Heppner TJ, Nelson MT (2001) Voltage dependence of the coupling of Ca2 + sparks to BKCa channels in urinary bladder smooth muscle. Am J Physiol-Cell Physiol 280:C481–C490. 10.1152/ajpcell.2001.280.3.C481 11171567 10.1152/ajpcell.2001.280.3.C481

[CR98] Hertz-Picciotto I, Schmidt RJ, Walker CK, Bennett DH, Oliver M, Shedd-Wise KM, LaSalle JM, Giulivi C, Puschner B, Thomas J, Roa DL, Pessah IN, Van de Water J, Tancredi DJ, Ozonoff S (2018) A prospective study of environmental exposures and early biomarkers in autism spectrum disorder: design, protocols, and preliminary data from the MARBLES study. Environ Health Perspect 126:117004. 10.1289/EHP535 30465702 10.1289/EHP535 PMC6371714

[CR99] Hoag N, Gani J (2015) Underactive bladder: clinical features, urodynamic parameters, and treatment. Int Neurourol J 19:185–189. 10.5213/inj.2015.19.3.185 26620901 10.5213/inj.2015.19.3.185 PMC4582091

[CR100] Hollywood MA, McCloskey KD, McHale NG, Thornbury KD (2000) Characterization of outward K + currents in isolated smooth muscle cells from sheep urethra. Am J Physiol-Cell Physiol 279:C420–C428. 10.1152/ajpcell.2000.279.2.C420 10913009 10.1152/ajpcell.2000.279.2.C420

[CR101] Horrigan FT, Aldrich RW (2002) Coupling between voltage sensor activation, Ca2 + binding and channel opening in large conductance (BK) potassium channels. J Gen Physiol 120:267–305. 10.1085/jgp.20028605 12198087 10.1085/jgp.20028605 PMC2229516

[CR102] Hotta S, Morimura K, Ohya S, Muraki K, Takeshima H, Imaizumi Y (2007) Ryanodine receptor type 2 deficiency changes excitation–contraction coupling and membrane potential in urinary bladder smooth muscle. J Physiol 582:489–506. 10.1113/jphysiol.2007.130302 17363382 10.1113/jphysiol.2007.130302 PMC2075324

[CR103] Hristov KL, Chen M, Kellett WF, Rovner ES, Petkov GV (2011) Large-conductance voltage- and Ca2+-activated K + channels regulate human detrusor smooth muscle function. American Journal of Physiology-Cell Physiology 301:C903. 10.1152/ajpcell.00495.2010 21697543 10.1152/ajpcell.00495.2010 PMC3191561

[CR104] Hristov KL, Afeli SAY, Parajuli SP, Cheng Q, Rovner ES, Petkov GV (2013) Neurogenic detrusor overactivity is associated with decreased expression and function of the large conductance voltage- and Ca2+-activated K + channels. PLoS One 8:e68052. 10.1371/journal.pone.0068052 23861849 10.1371/journal.pone.0068052 PMC3702577

[CR105] Hristov KL, Smith AC, Parajuli SP, Malysz J, Petkov GV (2014) Large-conductance voltage- and Ca2+-activated K + channel regulation by protein kinase C in Guinea pig urinary bladder smooth muscle. Am J Physiol-Cell Physiol 306:C460–C470. 10.1152/ajpcell.00325.2013 24352333 10.1152/ajpcell.00325.2013 PMC4098151

[CR106] Hristov KL, Parajuli SP, Provence A, Petkov GV (2016) Testosterone decreases urinary bladder smooth muscle excitability via novel signaling mechanism involving direct activation of the BK channels. Am J Physiol-Renal Physiol 311:F1253–F1259. 10.1152/ajprenal.00238.2016 27605581 10.1152/ajprenal.00238.2016 PMC5210203

[CR107] Hristov KL, Parajuli SP, Provence A, Rovner ES, Petkov GV (2017) Nongenomic modulation of the large conductance voltage- and Ca2+‐activated K + channels by estrogen: a novel regulatory mechanism in human detrusor smooth muscle. Physiol Rep 5:e13351. 10.14814/phy2.13351 28754781 10.14814/phy2.13351 PMC5532485

[CR108] Hsieh P-C, Chang Cshang-jen, Yang H-H, S.-D (2023) The differences in the adrenergic receptors of proximal urethra between sexes. Tzu Chi Med J Publish ahead Print. 10.4103/tcmj.tcmj_221_22 10.4103/tcmj.tcmj_221_22 PMC10399842 37545797

[CR256] Hu XQ, Xiao D, Zhu R, Huang X, Yang S, Wilson S, Zhang L (2011) Pregnancy upregulates large-conductance Ca(2+)-activated K(+) channel activity and attenuates myogenic tone in uterine arteries. Hypertension 58(6):1132–9. 10.1161/HYPERTENSIONAHA.111.179952. PMID: 22042813; PMCID: PMC3223357. 10.1161/HYPERTENSIONAHA.111.179952 PMC3223357 22042813

[CR109] Huang J, Chan C-K, Yee S, Deng Y, Bai Y, Chan SC, Tin MS, Liu X, Lok V, Zhang L, Xu W, Zheng Z-J, Teoh JY-C, Ng C-F, Wong MCS (2023) Global burden and temporal trends of lower urinary tract symptoms: a systematic review and meta-analysis. Prostate Cancer Prostatic Dis 26:421–428. 10.1038/s41391-022-00610-w 36414786 10.1038/s41391-022-00610-w

[CR110] Hudson DL, Guy AT, Fry P, O’Hare MJ, Watt FM, Masters JR (2001) Epithelial cell differentiation pathways in the human prostate: identification of intermediate phenotypes by keratin expression. J Histochem Cytochem 49:271–278. 10.1177/002215540104900214 11156695 10.1177/002215540104900214

[CR111] Imagawa T, Smith JS, Coronado R, Campbell KP (1987) Purified ryanodine receptor from skeletal muscle sarcoplasmic reticulum is the Ca2+-permeable pore of the calcium release channel. J Biol Chem 262:16636–16643 2445748

[CR112] Irwin DE, Kopp ZS, Agatep B, Milsom I, Abrams P (2011) Worldwide prevalence estimates of lower urinary tract symptoms, overactive bladder, urinary incontinence and bladder outlet obstruction. BJU Int 108:1132–1138. 10.1111/j.1464-410X.2010.09993.x 21231991 10.1111/j.1464-410X.2010.09993.x

[CR113] Jarvis TR, Chughtai B, Kaplan SA (2015) Testosterone and benign prostatic hyperplasia. Asian J Androl 17:212–216. 10.4103/1008-682X.140966 25337845 10.4103/1008-682X.140966 PMC4650459

[CR114] Jensen BS (2002) BMS-204352: a potassium channel opener developed for the treatment of stroke. CNS Drug Rev 8:353–360. 10.1111/j.1527-3458.2002.tb00233.x 12481191 10.1111/j.1527-3458.2002.tb00233.x PMC6741660

[CR115] Jeong SJ, Kim HJ, Lee YJ, Lee JK, Lee BK, Choo YM, Oh JJ, Lee SC, Jeong CW, Yoon CY, Hong SK, Byun S-S, Lee SE (2012) Prevalence and clinical features of detrusor underactivity among elderly with lower urinary tract symptoms: a comparison between men and women. Korean J Urol 53:342–348. 10.4111/kju.2012.53.5.342 22670194 10.4111/kju.2012.53.5.342 PMC3364474

[CR116] Jeong SJ, Lee M, Song SH, Kim H, Choo MS, Cho SY, Oh S-J, Study Group SEOUL (2021) Prevalence and urodynamic characteristics of detrusor overactivity with impaired contractility in the community-dwelling elderly with non-neurogenic lower urinary tract symptoms: is it from a single or two independent bladder dysfunctions? Investig Clin Urol 62:477. 10.4111/icu.20200471 34085790 10.4111/icu.20200471 PMC8246009

[CR117] Ji G, Barsotti RJ, Feldman ME, Kotlikoff MI (2002) Stretch-induced calcium release in smooth muscle. J Gen Physiol 119:533–543. 10.1085/jgp.20028514 12034761 10.1085/jgp.20028514 PMC2233869

[CR118] Ji G, Feldman ME, Greene KS, Sorrentino V, Xin H-B, Kotlikoff MI (2004) RYR2 proteins contribute to the formation of Ca(2+) sparks in smooth muscle. J Gen Physiol 123:377–386. 10.1085/jgp.200308999 15024040 10.1085/jgp.200308999 PMC2217466

[CR119] Jiang Z, Wallner M, Meera P, Toro L (1999) Human and rodent maxik channel β-subunit genes: cloning and characterization. Genomics 55:57–67. 10.1006/geno.1998.5627 9888999 10.1006/geno.1998.5627

[CR120] Jiang H-H, Song B, Lu G-S, Wen Q-J, Jin X-Y (2005) Loss of ryanodine receptor calcium-release channel expression associated with overactive urinary bladder smooth muscle contractions in a detrusor instability model. BJU Int 96:428–433. 10.1111/j.1464-410X.2005.05644.x 16042743 10.1111/j.1464-410X.2005.05644.x

[CR121] Jung J, Ahn HK, Huh Y (2012) Clinical and functional anatomy of the urethral sphincter. Int Neurourol J 16:102–106. 10.5213/inj.2012.16.3.102 23094214 10.5213/inj.2012.16.3.102 PMC3469827

[CR122] Kannan H, Radican L, Turpin RS, Bolge SC (2009) Burden of illness associated with lower urinary tract symptoms including overactive bladder/urinary incontinence. Urology 74:34–38. 10.1016/j.urology.2008.12.077 19428076 10.1016/j.urology.2008.12.077

[CR123] Kathrins M, Doersch K, Nimeh T, Canto A, Niederberger C, Seftel A (2016) The relationship between testosterone-replacement therapy and lower urinary tract symptoms: a systematic review. Urology 88:22–32. 10.1016/j.urology.2015.11.006 26616095 10.1016/j.urology.2015.11.006

[CR124] Kayigil Ö, Ahmed SI, Metin A (1999) The coexistence of intrinsic sphincter deficiency with type II stress incontinence. J Urol 162:1365–1366. 10.1016/S0022-5347(05)68289-4 10492198

[CR125] Keil Stietz KP, Kennedy CL, Sethi S, Valenzuela A, Nunez A, Wang K, Wang Z, Wang P, Spiegelhoff A, Puschner B, Bjorling DE, Lein PJ (2021) In utero and lactational PCB exposure drives anatomic changes in the juvenile mouse bladder. Curr Res Toxicol 2:1–18. 10.1016/j.crtox.2021.01.002 34337439 10.1016/j.crtox.2021.01.002 PMC8317607

[CR253] Kennedy CL, Spiegelhoff A, Wang K, Lavery T, Nunez A, Manuel R, Hillers-Ziemer L, Arendt LM, Stietz KPK (2021) The Bladder Is a Novel Target of Developmental Polychlorinated Biphenyl Exposure Linked to Increased Inflammatory Cells in the Bladder of Young Mice. Toxics 9(9):214. 10.3390/toxics9090214. PMID: 34564365; PMCID: PMC8473463. 10.3390/toxics9090214 PMC8473463 34564365

[CR252] Kennedy CL, Spiegelhoff A, Lavery T, Wang K, Manuel RS, Wang Z, Wildermuth H, Keil Stietz KP (2022) Developmental polychlorinated biphenyl (PCB) exposure alters voiding physiology in young adult male and female mice. Am J Clin Exp Urol 10(2):82–97. PMID: 35528463; PMCID: PMC9077147. PMC9077147 35528463

[CR126] Kerns JM, Damaser MS, Kane JM, Sakamoto K, Benson JT, Shott S, Brubaker L (2000) Effects of pudendal nerve injury in the female rat. Neurourol Urodyn 19:53–69. 10.1002/(sici)1520-6777(2000)19:1%3C53::aid-nau7%3E3.0.co;2-8 10602248 10.1002/(sici)1520-6777(2000)19:1<53::aid-nau7>3.0.co;2-8

[CR127] Kershen RT, Azadzoi KM, Siroky MB (2002) Blood flow, pressure and compliance in the male human bladder. J Urol 168:121–125. 10.1016/S0022-5347(05)64843-4 12050504

[CR128] Khan RN, Smith SK, Morrison JJ, Ashford MLJ (1997) Ca2 + dependence and pharmacology of large-conductance K + channels in nonlabor and labor human uterine myocytes. Am J Physiol-Cell Physiol 273:C1721–C1731. 10.1152/ajpcell.1997.273.5.C1721 10.1152/ajpcell.1997.273.5.C1721 9374660

[CR129] Kim KH, Bose DD, Ghogha A, Riehl J, Zhang R, Barnhart CD, Lein PJ, Pessah IN (2011) Para- and ortho-substitutions are key determinants of polybrominated diphenyl ether activity toward Ryanodine receptors and neurotoxicity. Environ Health Perspect 119:519–526. 10.1289/ehp.1002728 21106467 10.1289/ehp.1002728 PMC3080935

[CR130] King JT, Lovell PV, Rishniw M, Kotlikoff MI, Zeeman ML, McCobb DP (2006) β2 and β4 subunits of BK channels confer differential sensitivity to acute modulation by steroid hormones. J Neurophysiol 95:2878–2888. 10.1152/jn.01352.2005 16436475 10.1152/jn.01352.2005

[CR131] Kita M, Yunoki T, Takimoto K, Miyazato M, Kita K, de Groat WC, Kakizaki H, Yoshimura N (2010) Effects of bladder outlet obstruction on properties of Ca2+-activated K + channels in rat bladder. American Journal of Physiology-Regulatory, Integrative and Comparative Physiology 298:R1310–R1319. 10.1152/ajpregu.00523.2009 20200132 10.1152/ajpregu.00523.2009 PMC2867513

[CR132] Knaus HG, Schwarzer C, Koch RO, Eberhart A, Kaczorowski GJ, Glossmann H, Wunder F, Pongs O, Garcia ML, Sperk G (1996) Distribution of high-conductance Ca(2+)-activated K + channels in rat brain: targeting to axons and nerve terminals. J Neurosci Off J Soc Neurosci 16:955–963. 10.1523/JNEUROSCI.16-03-00955.1996 10.1523/JNEUROSCI.16-03-00955.1996 PMC6578788 8558264

[CR133] Kopač M (2024) Pediatric lower urinary tract dysfunction: a comprehensive exploration of clinical implications and diagnostic strategies. Biomedicines 12:945. 10.3390/biomedicines12050945 38790908 10.3390/biomedicines12050945 PMC11118197

[CR134] Koraitim MM (2008) The male urethral sphincter complex revisited: an anatomical concept and its physiological correlate. J Urol 179:1683–1689. 10.1016/j.juro.2008.01.010 18343449 10.1016/j.juro.2008.01.010

[CR135] Kozlova EV, Valdez MC, Denys ME, Bishay AE, Krum JM, Rabbani KM, Carrillo V, Gonzalez GM, Lampel G, Tran JD, Vazquez BM, Anchondo LM, Uddin SA, Huffman NM, Monarrez E, Olomi DS, Chinthirla BD, Hartman RE, Kodavanti PRS, Chompre G, Phillips AL, Stapleton HM, Henkelmann B, Schramm K-W, Curras-Collazo MC (2022) Persistent autism-relevant behavioral phenotype and social neuropeptide alterations in female mice offspring induced by maternal transfer of PBDE congeners in the commercial mixture DE-71. Arch Toxicol 96:335–365. 10.1007/s00204-021-03163-4 34687351 10.1007/s00204-021-03163-4 PMC8536480

[CR136] Kuijpers KAJ, Heesakkers JPFA, Schalken JA (2014) Alterations of the myovesical plexus of the human overactive detrusor. BioMed Res Int 2014:754596. 10.1155/2014/754596 24829917 10.1155/2014/754596 PMC4009145

[CR137] Kuiper GGJM, Carlsson B, Grandien K, Enmark E, Häggblad J, Nilsson S, Gustafsson J-Å (1997) Comparison of the ligand binding specificity and transcript tissue distribution of Estrogen receptors α and β. Endocrinology 138:863–870. https://doi.org/2020071619331713600 9048584 10.1210/endo.138.3.4979

[CR138] Kunisawa Y, Kawabe K, Niijima T, Honda K, Takenaka O (1985) A pharmacological study of alpha adrenergic receptor subtypes in smooth muscle of human urinary bladder base and prostatic urethra. J Urol. 10.1016/S0022-5347(17)47185-0 2991614 10.1016/s0022-5347(17)47185-0

[CR139] Kushnir A, Wajsberg B, Marks AR (2018) Ryanodine receptor dysfunction in human disorders. Biochimica et Biophysica Acta (BBA) 1865:1687–1697. 10.1016/j.bbamcr.2018.07.011 10.1016/j.bbamcr.2018.07.011 30040966

[CR140] Kyle BD (2014) Ion channels of the mammalian urethra. Channels (Austin) 8:393–401. 10.4161/19336950.2014.954224 25483582 10.4161/19336950.2014.954224 PMC4594508

[CR141] Kyle BD, Bradley E, Large R, Sergeant GP, McHale NG, Thornbury KD, Hollywood MA (2013) Mechanisms underlying activation of transient BK current in rabbit urethral smooth muscle cells and its modulation by IP_3_ -generating agonists. Am J Physiol-Cell Physiol 305:C609–C622. 10.1152/ajpcell.00025.2013 23804200 10.1152/ajpcell.00025.2013 PMC3761171

[CR142] Laborde EE, McVary KT (2009) Medical management of lower urinary tract symptoms. Rev Urol 11:S19–S25 20126608 PMC2812890

[CR143] Lacampagne A, Liu X, Reiken S, Bussiere R, Meli AC, Lauritzen I, Teich AF, Zalk R, Saint N, Arancio O, Bauer C, Duprat F, Briggs CA, Chakroborty S, Stutzmann GE, Shelanski ML, Checler F, Chami M, Marks AR (2017) Post-translational remodeling of Ryanodine receptor induces calcium leak leading to alzheimer’s disease-like pathologies and cognitive deficits. Acta Neuropathol (Berl) 134:749–767. 10.1007/s00401-017-1733-7 28631094 10.1007/s00401-017-1733-7

[CR144] Lai FA, Misra M, Xu L, Smith HA, Meissner G (1989) The ryanodine receptor-Ca2 + release channel complex of skeletal muscle sarcoplasmic reticulum. J Biol Chem 264:16776–16785. 10.1016/S0021-9258(19)84773-7 2550460

[CR145] Langton PD, Nelson MT, Huang Y, Standen NB (1991) Block of calcium-activated potassium channels in mammalian arterial myocytes by tetraethylammonium ions. Am J Physiol-Heart Circ Physiol 260:H927–H934. 10.1152/ajpheart.1991.260.3.H927 10.1152/ajpheart.1991.260.3.H927 1900393

[CR146] Lanner JT (2012) Ryanodine receptor physiology and its role in disease. In: Islam MS (ed) Calcium signaling. Springer Netherlands, Dordrecht, pp 217–234. 10.1007/978-94-007-2888-2_9 10.1007/978-94-007-2888-2_9 22453944

[CR250] Lavery TC, Spiegelhoff A, Wang K, Kennedy CL, Ridlon M, Keil Stietz KP (2023) Polychlorinated biphenyl (PCB) exposure in adult female mice can influence bladder contractility. Am J Clin Exp Urol 11(5):367–384. PMID: 37941647; PMCID: PMC10628623. PMC10628623 37941647

[CR147] Lee C-L, Kuo H-C (2017) Pathophysiology of benign prostate enlargement and lower urinary tract symptoms: current concepts. Tzu-Chi Med J 29:79–83. 10.4103/tcmj.tcmj_20_17 28757771 10.4103/tcmj.tcmj_20_17 PMC5509197

[CR148] Lee K, Isogai A, Antoh M, Kajioka S, Eto M, Hashitani H (2018) Role of K + channels in regulating spontaneous activity in the muscularis mucosae of Guinea pig bladder. Eur J Pharmacol 818:30–37. 10.1016/j.ejphar.2017.10.024 29050967 10.1016/j.ejphar.2017.10.024

[CR149] Lein PJ (2023) Chapter Four - Ryanodine receptor-dependent mechanisms of PCB developmental neurotoxicity, in: Kodavanti, P.R.S., Aschner, M., Costa, L.G. (Eds.), Advances in Neurotoxicology, Neurotoxicity of Halogenated Organic Compounds. Academic Press, pp. 137–178. 10.1016/bs.ant.2023.09.003 10.1016/bs.ant.2023.06.001 PMC10622110 37920427

[CR150] Lesiak A, Zhu M, Chen H, Appleyard SM, Impey S, Lein PJ, Wayman GA (2014) The environmental neurotoxicant PCB 95 promotes synaptogenesis via ryanodine receptor-dependent miR132 upregulation. J Neurosci 34:717–725. 10.1523/JNEUROSCI.2884-13.2014 24431430 10.1523/JNEUROSCI.2884-13.2014 PMC3891953

[CR151] Li Q, Yan J (2016) Modulation of BK channel function by auxiliary beta and gamma subunits. Int Rev Neurobiol 128:51–90. 10.1016/bs.irn.2016.03.015 27238261 10.1016/bs.irn.2016.03.015 PMC4918470

[CR152] Li L, Jiang C, Song B, Yan J, Pan J (2008) Altered expression of calcium-activated K and Cl channels in detrusor overactivity of rats with partial bladder outlet obstruction. BJU Int 101:1588–1594. 10.1111/j.1464-410X.2008.07522.x 18294303 10.1111/j.1464-410X.2008.07522.x

[CR153] Li Z, You M, Che X, Dai Y, Xu Y, Wang Y (2021) Perinatal exposure to BDE-47 exacerbated autistic-like behaviors and impairments of dendritic development in a valproic acid-induced rat model of autism. Ecotoxicol Environ Saf 212:112000. 10.1016/j.ecoenv.2021.112000 33550075 10.1016/j.ecoenv.2021.112000

[CR154] Li X, Hefti MM, Marek RF, Hornbuckle KC, Wang K, Lehmler H-J (2022) Assessment of polychlorinated biphenyls and their hydroxylated metabolites in postmortem human brain samples: age and brain region differences. Environ Sci Technol 56:9515–9526. 10.1021/acs.est.2c00581 35658127 10.1021/acs.est.2c00581 PMC9260965

[CR254] Li XT, Qiu XY (2015) 17β-Estradiol Upregulated Expression of α and β Subunits of Larger-Conductance Calcium-Activated K+ Channels (BK) via Estrogen Receptor β. J Mol Neurosci 56:799–807 10.1007/s12031-015-0502-0 10.1007/s12031-015-0502-0 25676031

[CR155] Linares V, Bellés M, Domingo JL (2015) Human exposure to PBDE and critical evaluation of health hazards. Arch Toxicol 89:335–356. 10.1007/s00204-015-1457-1 25637414 10.1007/s00204-015-1457-1

[CR156] Liu X, Betzenhauser MJ, Reiken S, Meli AC, Xie W, Chen B-X, Arancio O, Marks AR (2012) Role of leaky neuronal ryanodine receptors in stress-induced cognitive dysfunction. Cell 150:1055–1067. 10.1016/j.cell.2012.06.052 22939628 10.1016/j.cell.2012.06.052 PMC3690518

[CR157] Lorigo M, Mariana M, Lemos MC, Cairrao E (2020) Vascular mechanisms of testosterone: the non-genomic point of view. J Steroid Biochem Mol Biol 196:105496. 10.1016/j.jsbmb.2019.105496 31655180 10.1016/j.jsbmb.2019.105496

[CR158] Lyall K, Croen LA, Sjödin A, Yoshida CK, Zerbo O, Kharrazi M, Windham GC (2017) Polychlorinated biphenyl and organochlorine pesticide concentrations in maternal mid-pregnancy serum samples: association with autism spectrum disorder and intellectual disability. Environ Health Perspect 125:474–480. 10.1289/EHP277 27548254 10.1289/EHP277 PMC5332182

[CR159] Madhuvrata P, Singh M, Hasafa Z, Abdel-Fattah M (2012) Anticholinergic drugs for adult neurogenic detrusor overactivity: a systematic review and meta-analysis. Eur Urol 62:816–830. 10.1016/j.eururo.2012.02.036 22397851 10.1016/j.eururo.2012.02.036

[CR160] Malysz J, Petkov GV (2020) Urinary bladder smooth muscle ion channels: expression, function, and regulation in health and disease. Am J Physiol-Ren Physiol 319:F257–F283. 10.1152/ajprenal.00048.2020 10.1152/ajprenal.00048.2020 PMC7473901 32628539

[CR258] Martinez-Pinna J, Marroqui L, Hmadcha A, Lopez-Beas J, Soriano S, Villar-Pazos S, Alonso-Magdalena P, Dos Santos RS, Quesada I, Martin F, Soria B, Gustafsson JÅ, Nadal A (2019) Oestrogen receptor β mediates the actions of bisphenol-A on ion channel expression in mouse pancreatic beta cells. Diabetologia 62(9):1667–1680. 10.1007/s00125-019-4925-y. PMID: 31250031. 10.1007/s00125-019-4925-y 31250031

[CR161] Maserejian NN, Chen S, Chiu GR, Wager CG, Kupelian V, Araujo AB, McKinlay JB (2013) Incidence of lower urinary tract symptoms in a population-based study of men and women. Urology 82:560–564. 10.1016/j.urology.2013.05.009 23876577 10.1016/j.urology.2013.05.009 PMC3758799

[CR162] McDonough RC, Ryan ST (2016) Diagnosis and management of lower urinary tract dysfunction. Surg Clin North Am 96:441–452. 10.1016/j.suc.2016.02.003 27261787 10.1016/j.suc.2016.02.003

[CR163] Meera P, Wallner M, Toro L (2000) A neuronal β subunit (KCNMB4) makes the large conductance, voltage- and Ca2+-activated K + channel resistant to charybdotoxin and iberiotoxin. Proc Natl Acad Sci 97:5562–5567. 10.1073/pnas.100118597 10792058 10.1073/pnas.100118597 PMC25868

[CR164] Meissner G (1986) Ryanodine activation and inhibition of the Ca2 + release channel of sarcoplasmic reticulum. J Biol Chem 261:6300–6306. 10.1016/S0021-9258(19)84563-5 2422165

[CR165] Melman A, Bar-Chama N, McCullough A, Davies K, Christ G (2006) Hmaxi-K gene transfer in males with erectile dysfunction: results of the first human trial. Hum Gene Ther 17:1165–1176. 10.1089/hum.2006.17.1165 17134370 10.1089/hum.2006.17.1165

[CR166] Meredith AL, Thorneloe KS, Werner ME, Nelson MT, Aldrich RW (2004) Overactive bladder and incontinence in the absence of the BK large conductance Ca2+-activated K + channel. J Biol Chem 279:36746–36752. 10.1074/jbc.M405621200 15184377 10.1074/jbc.M405621200

[CR167] Michel MC, Vrydag W (2006) α1-, α2- and β-adrenoceptors in the urinary bladder, urethra and prostate. Br J Pharmacol 147:S88–S119. 10.1038/sj.bjp.0706619 16465187 10.1038/sj.bjp.0706619 PMC1751487

[CR168] Misonou H, Menegola M, Buchwalder L, Park EW, Meredith A, Rhodes KJ, Aldrich RW, Trimmer JS (2006) Immunolocalization of the Ca2+-activated K + channel Slo1 in axons and nerve terminals of mammalian brain and cultured neurons. J Comp Neurol 496:289–302. 10.1002/cne.20931 16566008 10.1002/cne.20931 PMC2605666

[CR169] Mouat JS, Li X, Neier K, Zhu Y, Mordaunt CE, La Merrill MA, Lehmler H-J, Jones MP, Lein PJ, Schmidt RJ, LaSalle JM (2023) Networks of placental DNA methylation correlate with maternal serum PCB concentrations and child neurodevelopment. Environ Res 220:115227. 10.1016/j.envres.2023.115227 36608759 10.1016/j.envres.2023.115227 PMC10518186

[CR170] Moussa M, Papatsoris A, Chakra MA, Fares Y, Dellis A (2020) Lower urinary tract dysfunction in common neurological diseases. Turk J Urol 46:S70–S78. 10.5152/tud.2020.20092 32384046 10.5152/tud.2020.20092 PMC7731959

[CR171] Mutoh S, Latifpour J, Saito M, Weiss RM (1997) Evidence for the presence of regional differences in the subtype specificity of muscarinic receptors in rabbit lower urinary tract. J Urol 157:717–721. 10.1016/S0022-5347(01)65257-1 8996405

[CR172] Myers JB, Mayer EN, Lenherr S (2016) Management options for sphincteric deficiency in adults with neurogenic bladder. Transl Androl Urol 5:145–157 26904420 10.3978/j.issn.2223-4683.2015.12.11 PMC4739985

[CR173] Nagahama K, Tsujii T, Morita T, Azuma H, Oshima H (1998) Differences between proximal and distal portions of the male rabbit posterior urethra in the physiological role of muscarinic cholinergic receptors. Br J Pharmacol 124:1175–1180. 10.1038/sj.bjp.0701952 9720788 10.1038/sj.bjp.0701952 PMC1565507

[CR257] Nagar D, Liu XT, Rosenfeld CR (2005) Estrogen regulates {beta}1-subunit expression in Ca(2+)-activated K(+) channels in arteries from reproductive tissues. Am J Physiol Heart Circ Physiol 289(4):H1417–27. 10.1152/ajpheart.01174.2004. PMID: 15923308. 10.1152/ajpheart.01174.2004 15923308

[CR174] Nardi A, Olesen S-P (2008) BK channel modulators: a comprehensive overview. Curr Med Chem 15:1126–1146. 10.2174/092986708784221412 18473808 10.2174/092986708784221412

[CR175] National Institutes of Health (NIH) (2015) Urologic Diseases Cost Americans $11 Billion a Year [WWW Document]. Natl. Inst. Health NIH. URL https://www.nih.gov/news-events/news-releases/urologic-diseases-cost-americans-11-billion-year (accessed 2.3.25)

[CR176] Neylon CB, Richards SM, Larsen MA, Agrotis A, Bobik A (1995) Multiple types of ryanodine receptor/Ca2 + release channels are expressed in vascular smooth muscle. Biochem Biophys Res Commun 215:814–821. 10.1006/bbrc.1995.2536 7488046 10.1006/bbrc.1995.2536

[CR247] Ng M, Leslie SW, Baradhi KM. Benign Prostatic Hyperplasia (2025) [Updated 2024 Oct 20]. In: StatPearls [Internet]. Treasure Island (FL): StatPearls Publishing; 2025 Jan. https://www.ncbi.nlm.nih.gov/books/NBK558920/32644346

[CR177] Nicholson TM, Ricke EA, Marker PC, Miano JM, Mayer RD, Timms BG, vom Saal FS, Wood RW, Ricke WA (2012) Testosterone and 17β-estradiol induce glandular prostatic growth, bladder outlet obstruction, and voiding dysfunction in male mice. Endocrinology 153:5556–5565 22948219 10.1210/en.2012-1522 PMC3473198

[CR255] Nishimura I, Ui-Tei K, Saigo K, Ishii H, Sakuma Y, Kato M (2008) 17beta-estradiol at physiological concentrations augments Ca(2+) -activated K+ currents via estrogen receptor beta in the gonadotropin-releasing hormone neuronal cell line GT1-7. Endocrinology 149(2):774–82. 10.1210/en.2007-0759. PMID: 17962348. 10.1210/en.2007-0759 17962348

[CR249] Niwa S, Ohya S, Kojima Y, Sasaki S, Yamamura H, Sakuragi M, Kohri K, Imaizumi Y (2012) Down-regulation of the large-conductance Ca(2+)-activated K+ channel, K(Ca)1.1 in the prostatic stromal cells of benign prostate hyperplasia. Biol Pharm Bull 35(5):737–44. 10.1248/bpb.35.737. PMID: 22687410. 10.1248/bpb.35.737 22687410

[CR246] Norton PA (1996) Etiology of genuine stress incontinence. In: Brubaker LT, Saclarides TJ (eds The female pelvic floor: disorders of function and support. Davis, Philadelphia, pp153–157

[CR178] Norton P, Brubaker L (2006) Urinary incontinence in women. Lancet Lond Engl 367:57–67. 10.1016/S0140-6736(06)67925-7 10.1016/S0140-6736(06)67925-7 16399154

[CR179] Oesterling JE (1994) Endocrine therapies for symptomatic benign prostatic hyperplasia. Urology 43:7–16. 10.1016/0090-4295(94)90212-7 7509536 10.1016/0090-4295(94)90212-7

[CR180] Ohi Y, Yamamura H, Nagano N, Ohya S, Muraki K, Watanabe M, Imaizumi Y (2001) Local Ca2 + transients and distribution of BK channels and ryanodine receptors in smooth muscle cells of guinea-pig Vas deferens and urinary bladder. J Physiol 534:313. 10.1111/j.1469-7793.2001.t01-3-00313.x 11454953 10.1111/j.1469-7793.2001.t01-3-00313.x PMC2278703

[CR181] Osman NI, Chapple CR, Abrams P, Dmochowski R, Haab F, Nitti V, Koelbl H, van Kerrebroeck P, Wein AJ (2014) Detrusor underactivity and the underactive bladder: A new clinical entity?? A review of current terminology, definitions, epidemiology, aetiology, and diagnosis. Eur Urol 65:389–398. 10.1016/j.eururo.2013.10.015 24184024 10.1016/j.eururo.2013.10.015

[CR182] Otsu K, Willard HF, Khanna VK, Zorzato F, Green NM, MacLennan DH (1990) Molecular cloning of cDNA encoding the Ca2 + release channel (ryanodine receptor) of rabbit cardiac muscle sarcoplasmic reticulum. J Biol Chem 265:13472–13483. 10.1016/S0021-9258(18)77371-7 2380170

[CR183] Panesar HK, Kennedy CL, Stietz K, Lein KP, P.J (2020) Polychlorinated biphenyls (PCBs): risk factors for autism. Spectr Disorder? Toxics 8:70. 10.3390/toxics8030070 10.3390/toxics8030070 PMC7560399 32957475

[CR184] Parajuli SP, Petkov GV (2013) Activation of muscarinic M3 receptors inhibits large-conductance voltage- and Ca2+-activated K + channels in rat urinary bladder smooth muscle cells. Am J Physiol-Cell Physiol 305:C207–C214. 10.1152/ajpcell.00113.2013 23703523 10.1152/ajpcell.00113.2013 PMC3725628

[CR185] Parajuli SP, Hristov KL, Cheng Q, Malysz J, Rovner ES, Petkov GV (2015) Functional link between muscarinic receptors and large-conductance Ca2+-activated K + channels in freshly isolated human detrusor smooth muscle cells. Pflüg Arch - Eur J Physiol 467:665–675. 10.1007/s00424-014-1537-8 10.1007/s00424-014-1537-8 PMC4247359 24867682

[CR186] Petkov GV, Nelson MT (2005) Differential regulation of Ca2+-activated K + channels by beta-adrenoceptors in Guinea pig urinary bladder smooth muscle. Am J Physiol Cell Physiol 288:C1255–1263. 10.1152/ajpcell.00381.2004 15677377 10.1152/ajpcell.00381.2004

[CR187] Porcari I, Uccella S, Casprini C, Bosco M, Zorzato PC, Garzon S (2025) Vulvovaginal estrogen therapy for urinary symptoms in postmenopausal women: a review and meta-analysis. Climacteric 0:1–10. 10.1080/13697137.2025.2517138 10.1080/13697137.2025.2517138 40569036

[CR188] Provence A, Hristov KL, Parajuli SP, Petkov GV (2015) Regulation of Guinea pig detrusor smooth muscle excitability by 17β-estradiol: the role of the large conductance voltage- and Ca2+-activated K + channels. PLoS One 10:e0141950. 10.1371/journal.pone.0141950 26536038 10.1371/journal.pone.0141950 PMC4633058

[CR189] Resnik E, Herron J, Fu R, Ivy DD, Cornfield DN (2006) Oxygen tension modulates the expression of pulmonary vascular BKCa channel α- and β-subunits. Am J Physiol-Lung Cell Mol Physiol 290:L761–L768. 10.1152/ajplung.00283.2005 16284215 10.1152/ajplung.00283.2005

[CR190] Reynolds WS, Fowke J, Dmochowski R (2016) The burden of overactive bladder on US public health. Curr Bladder Dysfunct Rep 11:8–13. 10.1007/s11884-016-0344-9 27057265 10.1007/s11884-016-0344-9 PMC4821440

[CR251] Ridlon M, Spiegelhoff A, Kennedy CL, Lavery T, Wang K, Tlapa J, Jordan T, Tanaka LF, Stietz KK (2025) Developmental polychlorinated biphenyl (PCB) exposure impacts on voiding physiology persist into adulthood and influence sensitivity to bladder stimuli in mice. Curr Res Toxicol 8:100227. 10.1016/j.crtox.2025.100227. PMID: 40144452; PMCID: PMC11937689. 10.1016/j.crtox.2025.100227 PMC11937689 40144452

[CR248] Roehrborn CG (2005) Benign prostatic hyperplasia: an overview. Rev Urol. 7 Suppl 9(Suppl 9):S3–S14. PMID: 16985902; PMCID: PMC1477638. PMC1477638 16985902

[CR191] Rosenberg J, Byrtus M, Stengl M (2016) Original research: combined model of bladder detrusor smooth muscle and interstitial cells. Exp Biol Med 241:1853–1864. 10.1177/1535370216655402 10.1177/1535370216655402 PMC5027948 27328937

[CR192] Rother P, Löffler S, Dorschner W, Reibiger I, Bengs T (1996) Anatomic basis of micturition and urinary continence. Muscle systems in urinary bladder neck during ageing. Surg Radiol Anat 18:173–177. 10.1007/BF02346123 8873329 10.1007/BF02346123

[CR193] Rottgen TS, Fancher IS, Asano S, Widlanski TS, Dick GM (2014) Bisphenol A activates BK channels through effects on α and β1 subunits. Channels Austin Tex 8:249–257. 10.4161/chan.27709 24476761 10.4161/chan.27709 PMC4203754

[CR194] Rubin EB, Buehler AE, Halpern SD (2016) States worse than death among hospitalized patients with serious illnesses. JAMA Intern Med 176:1557–1559. 10.1001/jamainternmed.2016.4362 27479808 10.1001/jamainternmed.2016.4362 PMC6848972

[CR195] Ruetten H, Wegner KA, Zhang HL, Wang P, Sandhu J, Sandhu S, Mueller B, Wang Z, Macoska J, Peterson RE, Bjorling DE, Ricke WA, Marker PC, Vezina CM (2019) Impact of sex, androgens, and prostate size on C57BL/6J mouse urinary physiology: functional assessment. Am J Physiol-Ren Physiol 317:F996–F1009. 10.1152/ajprenal.00270.2019 10.1152/ajprenal.00270.2019 PMC6843040 31390231

[CR245] Salazar BH, Hoffman KA, Zhang C, Zhang Y, Cruz Y, Boone TB, Munoz A (2019) Modulatory effects of intravesical P2X2/3 purinergic receptor inhibition on lower urinary tract electromyographic properties and voiding function of female rats with moderate or severe spinal cord injury. BJU Int 123:538–547. 10.1111/bju.14561 10.1111/bju.14561 PMC6715153 30255543

[CR196] Sancho M, Kyle BD (2021) The large-conductance, calcium-activated potassium channel: a big key regulator of cell physiology. Front Physiol 12:750615. 10.3389/fphys.2021.750615 34744788 10.3389/fphys.2021.750615 PMC8567177

[CR197] Sanderson JT (2006) The steroid hormone biosynthesis pathway as a target for endocrine-disrupting chemicals. Toxicol Sci 94:3–21. 10.1093/toxsci/kfl051 16807284 10.1093/toxsci/kfl051

[CR198] Satoh H, Mori K, Furuhama K (2001) Morphological and immunohistochemical characteristics of the heterogeneous Prostate-like glands (Paraurethral Gland) seen in female Brown-Norway rats. Toxicol Pathol 29:237–241. 10.1080/019262301317052512 11421491 10.1080/019262301317052512

[CR199] Sausbier U, Sausbier M, Sailer CA, Arntz C, Knaus H-G, Neuhuber W, Ruth P (2006) Ca2+ -activated K + channels of the BK-type in the mouse brain. Histochem Cell Biol 125:725–741. 10.1007/s00418-005-0124-7 16362320 10.1007/s00418-005-0124-7

[CR200] Sergeant GP, Hollywood MA, McCloskey KD, Thornbury KD, McHale NG (2000) Specialised pacemaking cells in the rabbit urethra. J Physiol 526:359–366. 10.1111/j.1469-7793.2000.t01-2-00359.x 10896724 10.1111/j.1469-7793.2000.t01-2-00359.x PMC2270007

[CR201] Sergeant GP, Hollywood MA, Thornbury KD (2019) Spontaneous activity in urethral smooth muscle. Adv Exp Med Biol 1124:149–167. 10.1007/978-981-13-5895-1_6 31183826 10.1007/978-981-13-5895-1_6

[CR202] Serysheva II (2004) Structural insights into excitation—contraction coupling by electron cryomicroscopy. Biochem Mosc 69:1226–1232. 10.1007/PL00021759 10.1007/s10541-005-0068-5 15627376

[CR203] Sethi S, Keil KP, Lein PJ (2018) 3,3’-dichlorobiphenyl (PCB 11) promotes dendritic arborization in primary rat cortical neurons via a CREB-dependent mechanism. Arch Toxicol 92:3337–3345. 10.1007/s00204-018-2307-8 30225637 10.1007/s00204-018-2307-8 PMC6196112

[CR204] Sethi S, Morgan RK, Feng W, Lin Y, Li X, Luna C, Koch M, Bansal R, Duffel MW, Puschner B, Zoeller RT, Lehmler H-J, Pessah IN, Lein PJ (2019) Comparative analyses of the 12 most abundant PCB congeners detected in human maternal serum for activity at the thyroid hormone receptor and ryanodine receptor. Environ Sci Technol 53:3948–3958. 10.1021/acs.est.9b00535 30821444 10.1021/acs.est.9b00535 PMC6457253

[CR205] Sethi S, Stietz K, Valenzuela KP, Klocke AE, Silverman CR, Puschner JL, Pessah B, Lein IN (2021) Developmental exposure to a human-relevant polychlorinated biphenyl mixture causes behavioral phenotypes that vary by sex and genotype in juvenile mice expressing human mutations that modulate neuronal calcium. Front Neurosci 15:766826. 10.3389/fnins.2021.766826 34938155 10.3389/fnins.2021.766826 PMC8685320

[CR206] Siddiqi MA, Laessig RH, Reed KD (2003) Polybrominated diphenyl ethers (PBDEs): new pollutants-old diseases. Clin Med Res 1:281–290 15931321 10.3121/cmr.1.4.281 PMC1069057

[CR207] Song R, Hu X-Q, Romero M, Holguin MA, Kagabo W, Xiao D, Wilson SM, Zhang L (2021) Ryanodine receptor subtypes regulate Ca2 + sparks/spontaneous transient outward currents and myogenic tone of uterine arteries in pregnancy. Cardiovasc Res 117:792–804. 10.1093/cvr/cvaa089 32251501 10.1093/cvr/cvaa089 PMC7898951

[CR208] Sprossmann F, Pankert P, Sausbier U, Wirth A, Zhou X-B, Madlung J, Zhao H, Bucurenciu I, Jakob A, Lamkemeyer T, Neuhuber W, Offermanns S, Shipston MJ, Korth M, Nordheim A, Ruth P, Sausbier M (2009) Inducible knockout mutagenesis reveals compensatory mechanisms elicited by constitutive BK channel deficiency in overactive murine bladder. FEBS J 276:1680–1697. 10.1111/j.1742-4658.2009.06900.x 19220851 10.1111/j.1742-4658.2009.06900.x PMC4025950

[CR209] Stewart WF, Van Rooyen JB, Cundiff GW, Abrams P, Herzog AR, Corey R, Hunt TL, Wein AJ (2003) Prevalence and burden of overactive bladder in the united States. World J Urol 20:327–336. 10.1007/s00345-002-0301-4 12811491 10.1007/s00345-002-0301-4

[CR210] Stoddard N, Leslie SW (2025) Histology, male urethra. StatPearls. StatPearls Publishing, Treasure Island (FL) 31194395

[CR211] Su HI, Freeman EW (2009) Hormone changes associated with the menopausal transition. Minerva Ginecol 61:483–489 19942836 PMC3823936

[CR212] Sun B, Yao J, Ni M, Wei J, Zhong X, Guo W, Zhang L, Wang R, Belke D, Chen Y-X, Lieve KVV, Broendberg AK, Roston TM, Blankoff I, Kammeraad JA, Von Alvensleben JC, Lazarte J, Vallmitjana A, Bohne LJ, Rose RA, Benitez R, Hove-Madsen L, Napolitano C, Hegele RA, Fill M, Sanatani S, Wilde AAM, Roberts JD, Priori SG, Jensen HK, Chen SRW (2021) Cardiac ryanodine receptor calcium release deficiency syndrome. Sci Transl Med 13:eaba7287. 10.1126/scitranslmed.aba7287 33536282 10.1126/scitranslmed.aba7287

[CR213] Tahra A, Bayrak Ö, Dmochowski R (2022) The epidemiology and population-based studies of women with lower urinary tract symptoms: a systematic review. Turk J Urol 48:155–165. 10.5152/tud.2022.21325 35420059 10.5152/tud.2022.21325 PMC9612779

[CR244] Teramoto N, Brading AF (1996) Activation by levcromakalim and metabolic inhibition of glibenclamide-sensitive K channels in smooth muscle cells of pig proximal urethra. Br J Pharmacol 118:635–642. 10.1111/j.1476-5381.1996.tb15448.x 10.1111/j.1476-5381.1996.tb15448.x PMC1909732 8762088

[CR214] Thomas S, Dunn CD, Campbell LJ, Strand DW, Vezina CM, Bjorling DE, Penniston KL, Li L, Ricke WA, Goldberg TL (2021) A multi-omic investigation of male lower urinary tract symptoms: potential role for JC virus. PLoS One 16:e0246266. 10.1371/journal.pone.0246266 33630889 10.1371/journal.pone.0246266 PMC7906371

[CR215] Thorneloe KS, Meredith AL, Knorn AM, Aldrich RW, Nelson MT (2005) Urodynamic properties and neurotransmitter dependence of urinary bladder contractility in the BK channel deletion model of overactive bladder. Am J Physiol-Ren Physiol 289:F604–F610. 10.1152/ajprenal.00060.2005 10.1152/ajprenal.00060.2005 15827347

[CR216] Todd JJ, Lawal TA, Chrismer IC, Kokkinis A, Grunseich C, Jain MS, Waite MR, Biancavilla V, Pocock S, Brooks K, Mendoza CJ, Norato G, Cheung K, Riekhof W, Varma P, Colina-Prisco C, Emile-Backer M, Meilleur KG, Marks AR, Webb Y, Marcantonio EE, Foley AR, Bönnemann CG, Mohassel P (2024) Rycal S48168 (ARM210) for RYR1-related myopathies: a phase one, open-label, dose-escalation trial. EClin Med 68:102433. 10.1016/j.eclinm.2024.102433 10.1016/j.eclinm.2024.102433 PMC10839573 38318125

[CR217] Torres YP, Granados ST, Latorre R (2014) Pharmacological consequences of the coexpression of BK channel Î± and auxiliary Î² subunits. Front Physiol 5:383. 10.3389/fphys.2014.00383 25346693 10.3389/fphys.2014.00383 PMC4193333

[CR218] Tran EL, Stuedemann SA, Ridlon M, Link OD, Stietz K, Crawford KP, L.K (2025) Genetic tools that target mechanoreceptors produce reliable labeling of bladder afferents and altered mechanosensation. Am J Physiol -Ren Physiol 328:F360–F374. 10.1152/ajprenal.00151.2024 10.1152/ajprenal.00151.2024 PMC12192779 39611874

[CR219] Ueda S, Satake N, Shibata S (1984) Alpha 1- and alpha 2-adrenoceptors in the smooth muscle of isolated rabbit urinary bladder and urethra. Eur J Pharmacol 103:249–254. 10.1016/0014-2999(84)90484-9 6149137 10.1016/0014-2999(84)90484-9

[CR220] Uvelius B, Gabella G (1980) Relation between cell length and force production in urinary bladder smooth muscle. Acta Physiol Scand 110:357–365. 10.1111/j.1748-1716.1980.tb06681.x 7234441 10.1111/j.1748-1716.1980.tb06681.x

[CR221] Valente S, DuBeau C, Chancellor D, Okonski J, Vereecke A, Doo F, Lajiness M, Diokno A, Chancellor M (2014) Epidemiology and demographics of the underactive bladder: a cross-sectional survey. Int Urol Nephrol 46:7–10. 10.1007/s11255-014-0811-1 10.1007/s11255-014-0811-1 25238889

[CR222] Valverde MA, Rojas P, Amigo J, Cosmelli D, Orio P, Bahamonde MI, Mann GE, Vergara C, Latorre R (1999) Acute activation of Maxi-K channels (hSlo) by estradiol binding to the β subunit. Science 285:1929–1931. 10.1126/science.285.5435.1929 10489376 10.1126/science.285.5435.1929

[CR223] van Koeveringe Ga, Vahabi B, Andersson Ke, Kirschner-Herrmans R, Oelke M (2011) Detrusor underactivity: A plea for new approaches to a common bladder dysfunction. Neurourol Urodyn 30:723–728. 10.1002/nau.21097 21661020 10.1002/nau.21097

[CR224] Vaughan CP, Markland AD, McGwin G, Lukacz ES, Brady SS, Lacoursiere YD, Wyman JF, Sutcliffe S, Smith AL, Kenton K, Stapleton A, Brubaker L, Harlow BL (2025) Association of menopausal status and hormone use with bladder health and lower urinary tract symptoms in US women: results from the RISE FOR HEALTH study. Menopause N Y 32:583–591. 10.1097/GME.0000000000002541 10.1097/GME.0000000000002541 PMC12395369 40298786

[CR225] Vuong AM, Yolton K, Dietrich KN, Braun JM, Lanphear BP, Chen A (2018) Exposure to polybrominated diphenyl ethers (PBDEs) and child behavior: current findings and future directions. Horm Behav Endocr Disrupting Chemicals Behav 101:94–104. 10.1016/j.yhbeh.2017.11.008 10.1016/j.yhbeh.2017.11.008 29137973

[CR226] Wakle-Prabagaran M, Lorca RA, Ma X, Stamnes SJ, Amazu C, Hsiao JJ, Karch CM, Hyrc KL, Wright ME, England SK (2016) BK_Ca_ channel regulates calcium oscillations induced by alpha-2-macroglobulin in human myometrial smooth muscle cells. Proc. Natl. Acad. Sci. 113. 10.1073/pnas.1516863113 10.1073/pnas.1516863113 PMC4843459 27044074

[CR227] Wang B, Rothberg BS, Brenner R (2006) Mechanism of β4 subunit modulation of BK channels. J Gen Physiol 127:449–465. 10.1085/jgp.200509436 16567466 10.1085/jgp.200509436 PMC2151511

[CR228] Wang B, Bugay V, Ling L, Chuang H-H, Jaffe DB, Brenner R (2016) Knockout of the BK β4-subunit promotes a functional coupling of BK channels and ryanodine receptors that mediate a fAHP-induced increase in excitability. J Neurophysiol 116:456–465. 10.1152/jn.00857.2015 27146987 10.1152/jn.00857.2015 PMC4978790

[CR229] Wang J, Ren L, Liu X, Liu J, Ling Q (2023) Underactive bladder and detrusor underactivity: new advances and prospectives. Int J Mol Sci 24:15517. 10.3390/ijms242115517 37958499 10.3390/ijms242115517 PMC10648240

[CR230] Wayman GA, Bose DD, Yang D, Lesiak A, Bruun D, Impey S, Ledoux V, Pessah IN, Lein PJ (2012) PCB-95 modulates the calcium-dependent signaling pathway responsible for activity-dependent dendritic growth. Environ Health Perspect 120:1003–1009. 10.1289/ehp.1104833 22534176 10.1289/ehp.1104833 PMC3404671

[CR231] Whitehead SA, Rice S (2006) Endocrine-disrupting chemicals as modulators of sex steroid synthesis. Best Pract Res Clin Endocrinol Metab Endocr Disrupters 20:45–61. 10.1016/j.beem.2005.09.003 10.1016/j.beem.2005.09.003 16522519

[CR232] Wong PW, Brackney WR, Pessah IN (1997) Ortho-substituted polychlorinated biphenyls alter microsomal calcium transport by direct interaction with Ryanodine receptors of mammalian brain. J Biol Chem 272:15145–15153. 10.1074/jbc.272.24.15145 9182535 10.1074/jbc.272.24.15145

[CR233] Wu X-R, Kong X-P, Pellicer A, Kreibich G, Sun T-T (2009) Uroplakins in urothelial biology, function and disease. Kidney Int 75:1153–1165. 10.1038/ki.2009.73 19340092 10.1038/ki.2009.73 PMC3717210

[CR234] Yaish I, Amir H, Eilam H, Gold R, Groutz A (2025) Effects of gender-affirming hormone therapy on lower urinary tract symptoms and sexual function among transgender individuals. Int J Gynecol Obstet 168:1292–1297. 10.1002/ijgo.15964 10.1002/ijgo.15964 PMC11823308 39400931

[CR235] Yamanishi T, Chapple CR, Chess-Williams R (2001) Which muscarinic receptor is important in the bladder? World J Urol 19:299–306. 10.1007/s003450100226 11760777 10.1007/s003450100226

[CR236] Yamanishi T, Kaga K, Fuse M, Shibata C, Uchiyama T (2015) Neuromodulation for the treatment of lower urinary tract symptoms. LUTS Low Urin Tract Symptoms 7:121–132. 10.1111/luts.12087 26663726 10.1111/luts.12087

[CR237] Yang C, Liu Z, Zeng L, Wu J, Zhang L (2025) Pharmacotherapy for children with central precocious puberty or early puberty: a systematic review and meta-analysis. Medicine (Baltimore) 104:e41936. 10.1097/MD.0000000000041936 40760548 10.1097/MD.0000000000041936 PMC12323909

[CR238] Yoshida M, Homma Y, Inadome A, Yono M, Seshita H, Miyamoto Y, Murakami S, Kawabe K, Ueda S (2001) Age-related changes in cholinergic and purinergic neurotransmission in human isolated bladder smooth muscles. Exp Gerontol 36:99–109. 10.1016/S0531-5565(00)00175-3 11162915 10.1016/s0531-5565(00)00175-3

[CR239] Yu J, Chae MR, Han DH, Kang SJ, Shin J, Sung HH (2025) Acute dual therapeutic effects of the BKCa channel opener LDD175 on erectile dysfunction and lower urinary tract symptoms in chronic pelvic ischemia: a preliminary study. Asian J Androl. 10.4103/aja202522 40405356 10.4103/aja202522 PMC12637880

[CR240] Zaviacic M, Danihel L, Ruzickova M, Blazekova J, Itoh Y, Okutani R, Kawai T (1997) Immunohistochemical localization of human protein 1 in the female prostate (Skene’s gland) and the male prostate. Histochem J 29:219–227. 10.1023/A:1026401909678 9472384 10.1023/a:1026401909678

[CR241] Zhang C, Chen Y, Yin L, Deng G, Xia X, Tang X, Zhang Y, Yan J (2024) Investigating the impact of estrogen levels on voiding characteristics, bladder structure, and related proteins in a mouse model of menopause-induced lower urinary tract symptoms. Biomolecules 14:1044. 10.3390/biom14091044 39334811 10.3390/biom14091044 PMC11429749

[CR242] Zheng J, Zhou H, Yang M, Song S, Dai Q, Ji G, Zhou Z (2020) Reduced Ca2 + spark activity contributes to detrusor overactivity of rats with partial bladder outlet obstruction. Aging 12:4163–4177. 10.18632/aging.102855 32112553 10.18632/aging.102855 PMC7093189

